# The N-Terminal Region of *Drosophila* MSL1 Interacts with Zinc Finger Proteins and Promotes the Recruitment of the MSL Complex to the X Chromosome in Males

**DOI:** 10.3390/ijms27219861

**Published:** 2026-11-03

**Authors:** Valentin Babosha, Varvara Ryzhkova, Anastasia Revel-Muroz, Evgeniya Tikhonova, Yulia Vasileva, Oksana Maksimenko, Pavel Georgiev

**Affiliations:** 1 Department of the Control of Genetic Processes, Institute of Gene Biology, Russian Academy of Sciences, 34/5 Vavilova Str., 119334 Moscow, Russia; v.babosha@gmail.com (V.B.); varvara.e.ryzhkova@gmail.com (V.R.); fall2987@mail.ru (E.T.); vasileva.mcb@gmail.com (Y.V.); 2 Center for Genome Research, Institute of Gene Biology, Russian Academy of Sciences, 34/5 Vavilova Str., 119334 Moscow, Russia; revelanastasia@gmail.com

**Keywords:** architectural C2H2 proteins, zinc finger C2H2 proteins, dosage compensation, transcription factor, MSL2, CLAMP, coiled-coil domain

## Abstract

The *Drosophila* protein male-specific lethal 1 (MSL1) is part of the five-subunit male-specific lethal (MSL) complex that binds only to the X chromosome in males. MSL1 contains a 93-amino-acid N-terminal unstructured region followed by a coiled-coil domain that forms a homodimer. The MSL1 dimer and male-specific lethal 2 (MSL2), which interacts with its coiled-coil domain, form a core that is primarily responsible for the specific recruitment of the MSL complex to X-chromosome high-affinity sites (HASs). In a yeast two-hybrid analysis, we found that the N-terminal region of MSL1, primarily amino acids 66–85 and the coiled-coil domain, interacts with many DNA-binding transcription factors. The coiled-coil domain of *Drosophila virilis* MSL1 (vCC) retains the ability to interact with *Drosophila melanogaster* MSL2 but not with zinc finger proteins. Expression of a double mutant of MSL1 (MSL1^Δ20vCC^), in which amino acids 66–85 (Δ20) have been deleted and the coiled-coil domain has been replaced with the vCC domain, significantly reduced dosage compensation in males due to decreased binding of the MSL complex to the X chromosome. Substitution of MSL1 with MSL1^vCC^ in females ectopically expressing MSL2 also significantly reduced recruitment of the MSL complex to the X chromosome. Altogether, these results indicate a key role of the N-terminal region of MSL1 not only in the assembly of the MSL complex but also in its recruitment to the X chromosome in males.

## 1. Introduction

It remains unknown how transcription regulatory complexes bind specifically to regions of chromatin that lack strong sequence specificity. The high-affinity association of transcription factors lacking DNA-binding domains with regulatory elements, primarily promoters, also remains poorly understood. The transcription factor male-specific lethal 1 (MSL1) is an interesting model protein for investigating these mechanisms. Although it lacks DNA-binding activity, it is associated with numerous gene promoters and forms part of the male-specific lethal (MSL) complex, which is specifically recruited to the X chromosome in *Drosophila* [1,2,3,4].

MSL1 comprises 1039 amino acid (aa) residues and contains two highly conserved domains [5,6]. The extended N-terminal coiled-coil domain (aa residues 94–176) is responsible for MSL1 dimerization [7,8]. One C-terminal domain of MSL1, referred to as PEHE, is required for interactions with male-specific lethal 3 (MSL3) and MOF [9,10,11], which together form a complex with acetylating activity [12,13]. MSL1 has independent functions in promoter activation [14,15].

In *Drosophila*, the primary function of MSL1 is dosage compensation, in which the expression of genes on the X chromosome is increased in males (X/Y) but not in females (X/X) to compensate for the presence of only one copy [2]. The multisubunit MSL complex, which binds only to the X chromosome in males [2,4,16], is responsible for the gene activation observed in dosage compensation. Dimerization of MSL1 via its coiled-coil domain is critical for the assembly of the MSL complex and its specific binding to the X chromosome in males [8]. The coiled-coil domains of MSL1 homodimers have been shown to interact with the N-terminal really interesting new gene (RING) domain and its flanking α-helices of two molecules of male-specific lethal 2 (MSL2) [8,17], which is expressed only in males [18]. The MSL3 and MOF proteins associate with the MSL complex exclusively through interaction with the PEHE domain of MSL1 [9,11]. Long non-coding RNAs on the X 1 (*roX1*, 3.7 kb) and 2 (*roX2*, 0.6 kb), which are expressed almost exclusively in males, perform a key but partially redundant function in the formation of the MSL complex [19]. *roX1* and *roX2* have been shown to increase the assembly efficiency of the MSL complex through specific interactions with its subunits [20,21,22]. In particular, efficient interaction between MSL1 and MSL2 requires specific interaction of roX RNAs with two regions of the unstructured N-terminal region of MSL1 [23]. Finally, maleless (MLE), an ATP-dependent RNA/DNA helicase, is incorporated into the MSL complex solely through interactions with *roX1* and *roX2* [24,25,26,27].

When MSL3 or MLE is inactivated, the MSL complex binds effectively only to a reproducible set of several hundred sites on the X chromosome, referred to as primary chromatin entry sites (CES) [28] or high-affinity sites (HAS) [29]. A GA-rich motif within the HAS/CES serves as an MSL recognition element (MRE) [28,29]. Structural analyses and an in vitro genome-wide DNA binding assay have shown that MSL2 specifically recognizes and binds to a subclass of HAS/CES, known as pioneering sites on the X (PionX), through its CXC and proline/basic-residue-rich domains [30,31,32].

The zinc finger protein chromatin-linked adaptor for MSL proteins (CLAMP) has been shown to be important for the specific recruitment of the MSL complex to the X chromosome in males [33,34]. CLAMP binds to GA-rich motifs in HAS/CES and helps organize open chromatin in these genomic regions [35]. CLAMP has also been shown to directly interact with MSL2 [35,36,37,38] and MLE [39]. Transcription from the 1.688^X^ satellite repeats [40] and pericentromeric 359 bp satellites [41] can partially compensate for the deletion of the *lncRNA:roX1* and *lncRNA:roX2* genes. Moreover, it has been suggested that small interfering RNAs (siRNAs) produced from 1.688^X^ satellite repeats participate in *Drosophila* dosage compensation [42].

Therefore, this study investigated the mechanisms by which MSL1 is recruited to gene promoters and its potential role in the MSL complex’s specific recognition of HAS/CES on the X chromosome in males. Since many transcription factors containing C2H2-type zinc finger domains (C2H2 proteins henceforth) are involved in the organization of active promoters [43,44,45,46,47,48,49,50,51], it also investigated whether MSL1 can directly interact with C2H2 proteins.

Yeast two-hybrid (Y2H) screening identified 18 C2H2 proteins that interact directly with the MSL1, which were subsequently confirmed in vivo using co-immunoprecipitation assays in *Drosophila melanogaster* Schneider 2 (S2) cells. Most of the identified C2H2 proteins were found to interact simultaneously with several regions in the unstructured N-terminal region and the adjacent coiled-coil domain of MSL1. Despite high conservation of the coiled-coil domain among *Drosophila* species, the MSL1 ortholog in *Drosophila virilis* fails to interact with C2H2 proteins from *D. melanogaster*, indicating species-specificity of such protein–protein interactions. In contrast, the coiled-coil domain of *D. virilis* MSL1 (vCC) retains the ability to interact with MSL2 from *D. melanogaster*.

The expression of an MSL1 mutant, MSL1^vCC^, in which the coiled-coil domain is replaced by the vCC domain, significantly reduced the efficiency of MSL complex recruitment to the X chromosome in females expressing MSL2. A similar but weaker effect was also observed with an MSL1 mutant lacking aa residues 66–85. In males, only the simultaneous deletion of aa residues 66–85 and replacement of the coiled-coil domain with the vCC domain in MSL1 has a strong effect on dosage compensation and recruitment of the MSL complex to the X chromosome. These results demonstrate that MSL1, along with MSL2, promotes the specific recruitment of the MSL complex to HASs/CESs.

## 2. Results

### 2.1. The N-Terminal Domain of MSL1 Interacts with Many C2H2 Proteins

**Figure 1:**
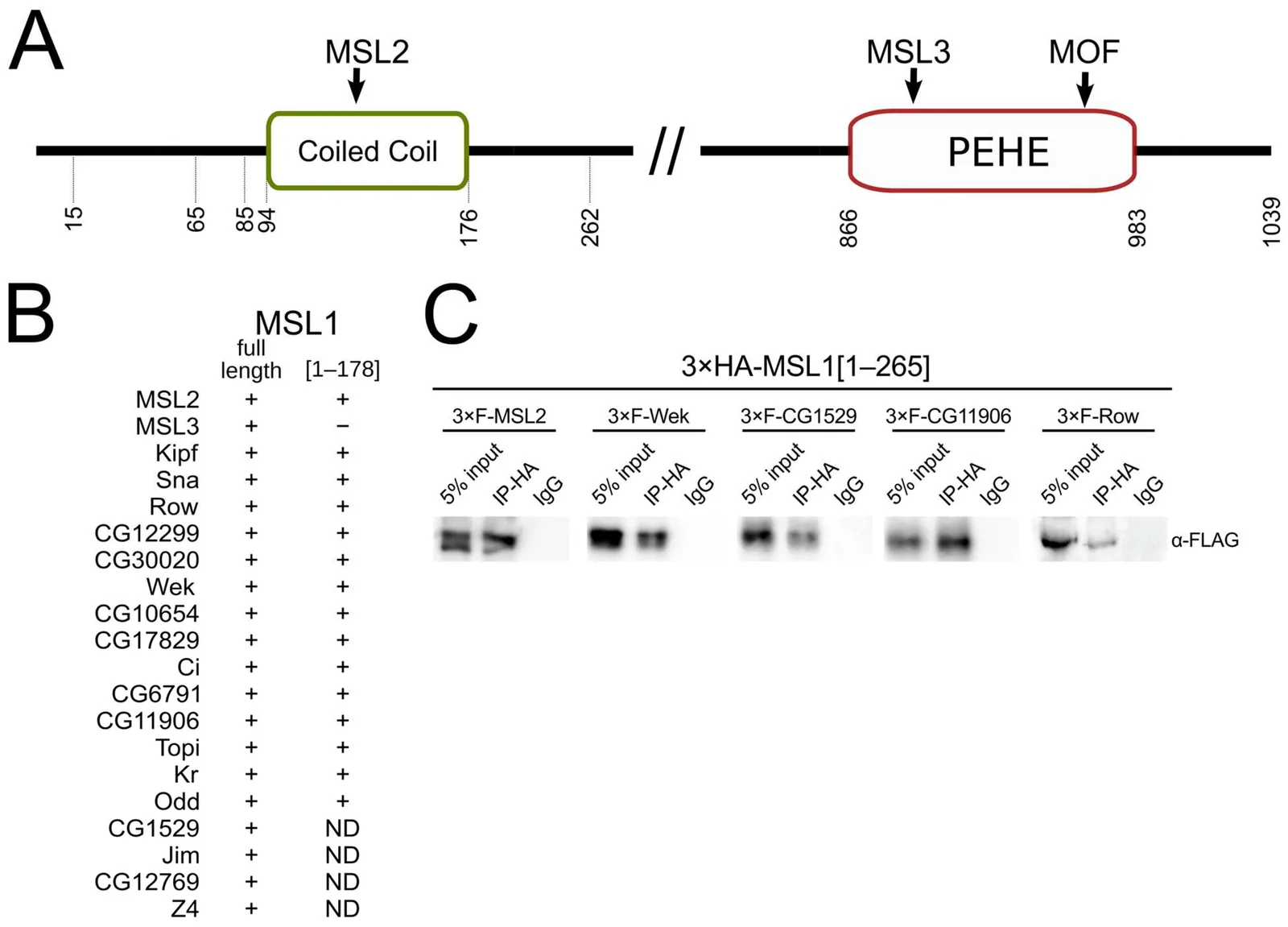
The interaction between MSL1 and C2H2 proteins. (**A**) A schematic representation of the MSL1 domains. coiled-coil (cc), domain interacting with MSL2; PEHE, domain interacting with MSL3 and MOF. (**B**) Interactions of full-length and truncated (aa residues 1–178) MSL1 with C2H2 proteins in the Y2H assay, with “+” denoting an interaction, and “−” denoting no interaction. MSL1 mutants were fused to the GAL4 DNA-binding domain and tested for interaction with C2H2 proteins fused to the GAL4 activating domain. (**C**) Co-immunoprecipitation of 3×HA-tagged MSL1[1–265] with 3×FLAG-tagged MSL2 (positive control), 3×FLAG-tagged Wek, 3×FLAG-tagged CG1529, 3×FLAG-tagged CG11906, and 3×FLAG-tagged Row. All tested proteins were co-expressed in *D. melanogaster* S2 cells. 3×HA-tagged MSL1[1–265] was immunoprecipitated with antibodies against the 3×HA epitope (IP-HA) and with nonspecific mouse immunoglobulin G (IgG, negative control). The immunoprecipitates were analyzed by Western blotting with anti-FLAG antibodies to detect the co-immunoprecipitated FLAG-tagged proteins. For both the experimental IP and the IgG control, 5% of the input and the entire immunoprecipitate (100% of the IP sample) were loaded.

The MSL1 protein (Figure 1A) contains a coiled-coil domain (aa residues 94–176), which is involved in MSL1 homodimerization and the interaction with MSL2, and a PEHE domain (aa residues 846–973), which binds to MSL3 and MOF [8,9,11,17]. To determine whether MSL1 can interact with C2H2 proteins, we employed a previously characterized library of 142 C2H2 proteins fused to the galactose-responsive transcription factor 4 (GAL4) activation domain (Table S1). We screened these C2H2 proteins against MSL1 fused to the GAL4 DNA-binding domain using Y2H assays in at least three biological replicates. We found MSL1 to interact with 18 C2H2 proteins (Figure 1B; Table S1), including activators (cubitus interruptus [Ci] and odd skipped [Odd]) and repressors (kruppel [Kr] and snail [Sna]) of early *Drosophila* development, as well as general transcription factors (putzig [Pzg/Z4] and relative of woc [Row]) and a protein (kipferl [Kipf]) involved in transposable element repression [52,53,54,55,56]. As a control, we also screened these C2H2 proteins against MSL2 using the same Y2H assay, identifying only an interaction with CLAMP (Table S1), which has been previously characterized [35,36], demonstrating the effectiveness of the Y2H assay.

Furthermore, we demonstrated interactions between the 14 identified C2H2 proteins and the 178-aa N-terminal region of MSL1 (termed MSL1[1–178]), which encompasses the unstructured region and the coiled-coil domain, using a Y2H assay (Figure 1B and Figure S1). To further demonstrate the significance of the Y2H results, we confirmed the interaction of 3×HA-tagged 265-aa N-terminal region of MSL1 (termed MSL1[1–265]) with four 3×FLAG-tagged C2H2 proteins, Wek, CG1529, CG11906, and Row, in a co-immunoprecipitation assay (Figure 1C).

### 2.2. Several Regions in the N-Terminal Region of MSL1 Simultaneously Interact with C2H2 Proteins

**Figure 2:**
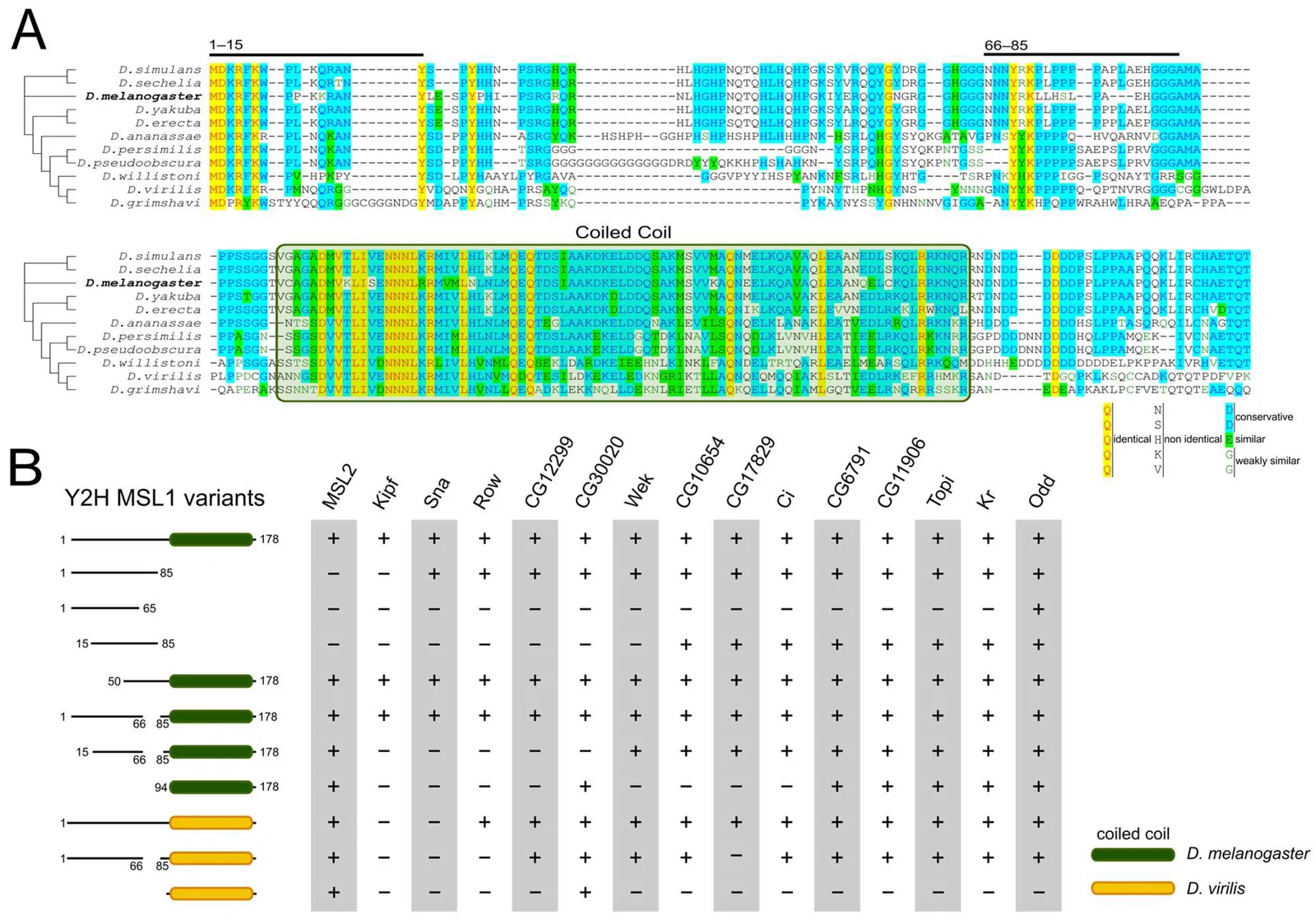
Mapping of the sequences in the N-terminal region of MSL1 involved in interactions with the identified C2H2 proteins. (**A**) Multiple sequence alignment of the N-terminal region of MSL1 [23], including the coiled-coil domain, among *Drosophilidae* species. The most important regions (aa residues 1–15 and 66–85, and the coiled-coil domain) in MSL1 for C2H2 protein interactions are indicated. *D. melanogaster* is highlighted in bold. (**B**) The contributions of N-terminal sequences in MSL1 to C2H2 protein interactions, as assessed in a Y2H assay, with “+” denoting an interaction, and “−” denoting no interaction. MSL1 mutants were fused to the GAL4 DNA-binding domain and tested for interaction with C2H2 proteins fused to the GAL4 activating domain.

The 178-aa N-terminal region of MSL1 contains sequences with varying degrees of conservation among *Drosophilidae* species (Figure 2A). The coiled-coil domain, which is required for interaction with MSL2, is most conserved; however, it demonstrates significant sequence changes across distant species, such as *D. melanogaster* and *D. virilis*. The first 15 aa residues correspond to the relatively conserved part of the N-terminal unstructured region of MSL1. Other sequences retain a high degree of homology only among related *Drosophila* species.

Our Y2H results demonstrated that the C2H2 proteins interact with MSL1[1–178], which includes the N-terminal unstructured region and the coiled-coil domain (Figure 1B). We used the Y2H assay to map key regions in MSL1[1–178] that determine interactions with 14 C2H2 proteins (Figure 2B and Figure S1). All proteins except Kipf interacted with aa residues 1–85 in MSL1[1–178]. In contrast, only Odd interacted with aa residues 1–65 in MSL1[1–178], suggesting that aa residues 66–85 may be involved in interactions with most C2H2 proteins. Additionally, deletion of aa residues 1–15 in MSL1[1–85] resulted in the loss of interaction with six C2H2 proteins (Kipf, Sna, Row, CG12299, CG30020, and Weckle [Wek]). Moreover, deletion of aa residues 1–15 or 66–85 in MSL1[1–178] had no visible effect on its interactions with C2H2 proteins. These results suggest that additional regions within MSL1[1–178] are involved in protein–protein interactions. Indeed, the coiled-coil domain (aa residues 94–176) supported interactions with only five C2H2 proteins in the Y2H assay (CG6791, CG11906, matotopetli [Topi], Kr, and Odd). The simultaneous deletion of aa residues 1–15 and 66–85 in MSL1[1–178] affected its interactions with only five C2H2 proteins (Kipf, Sna, Row, CG12299, and CG30020), suggesting the contribution of additional N-terminal sequences in MSL1 (aa residues 16–65 and 86–93) to protein–protein interactions with at least four remaining C2H2 proteins (Wek, CG10564, CG17829, and Ci).

The coiled-coil domains of *D. virilis* and *D. melanogaster* MSL1 exhibit relatively high homology (47.5%, Figure 2A). Therefore, we investigated whether the interaction with C2H2 proteins is conserved across the coiled-coil domains from *D. virilis* and *D. melanogaster* MSL1. In the Y2H assay, the vCC domain interacted with MSL2 but not with any of the examined C2H2 proteins (Figure 2B). Thus, during evolution, rapid adaptation likely occurred in interactions between C2H2 proteins and the coiled-coil domain of MSL1. A mutant of MSL1[1–178] in which the coiled-coil domain has been replaced by the vCC domain, MSL1[1–178]^vCC^, interacted with all C2H2 proteins except for Kipf and Sna. The additional deletion of aa residues 66–85 (Δ20) in MSL1[1–178]^vCC^ and MSL1[1–178]^Δ20vCC^ disrupted interactions with only CG17829 and Row. Comparing the interaction efficiency of the 1–65-aa region of MSL1 (interacts only with Odd) and MSL1[1–178]^Δ20vCC^ (interacts with 10 of the 14 C2H2 proteins), the 85–93-aa region appears to contribute to the stabilization of MSL1^Δ20vCC^ interactions with C2H2 proteins in the Y2H assay.

Altogether, the above results indicate that all MSL1[1–178] sequences are involved in interactions with C2H2 proteins to varying degrees. However, aa residues 1–15 and 66–85, as well as the coiled-coil domain, are the most important. Thus, according to our assumptions, the N-terminal part of MSL1 has three potential binding sites that simultaneously participate in numerous protein–protein interactions critical for MSL1 function. At the same time, different C2H2 proteins use different combinations of these sites, and the removal of any one of them, while at least one other site remains, may not have a visible effect. In previous work [23], we found that aa residues 1–15 and 41–65 are essential for specific interaction with *roX1* and *roX2* and are critical for the interaction between MSL1 and MSL2 in vivo. Thus, the unstructured N-terminus is simultaneously involved in numerous protein–protein and RNA–protein interactions that are critical for MSL1 function.

### 2.3. Simultaneously Replacing the Coiled-Coil Domain with the vCC Domain and Deleting aa Residues 66–85 in MSL1 Synergistically Impairs Dosage Compensation in D. melanogaster

To study the functional role of the N-terminal sequences of MSL1, we replaced the *msl-1* gene with an *attP* site in the *y^1^w^1118^* line using the CRISPR/Cas9 method [57,58,59]. The 1477 bp of the *msl-1* gene was substituted with the *attP* site and an Act5C:mCherry reporter flanked by *loxP* sites, resulting in the *msl-1^attP^* allele (Figure S2). The obtained deletions were balanced with *CyO*. Males homozygous for *msl-1^attP^* died predominantly at the larval stage, whereas females homozygous for *msl-1^attP^* (*msl-1^attP^*/*msl-1^attP^*) exhibited normal viability compared with those that were heterozygous (*msl-1^attP^*/*CyO*). Thus, we concluded that the resulting *msl-1^attP^*/*CyO* line does not harbor non-target mutations that affect viability.

**Figure 3:**
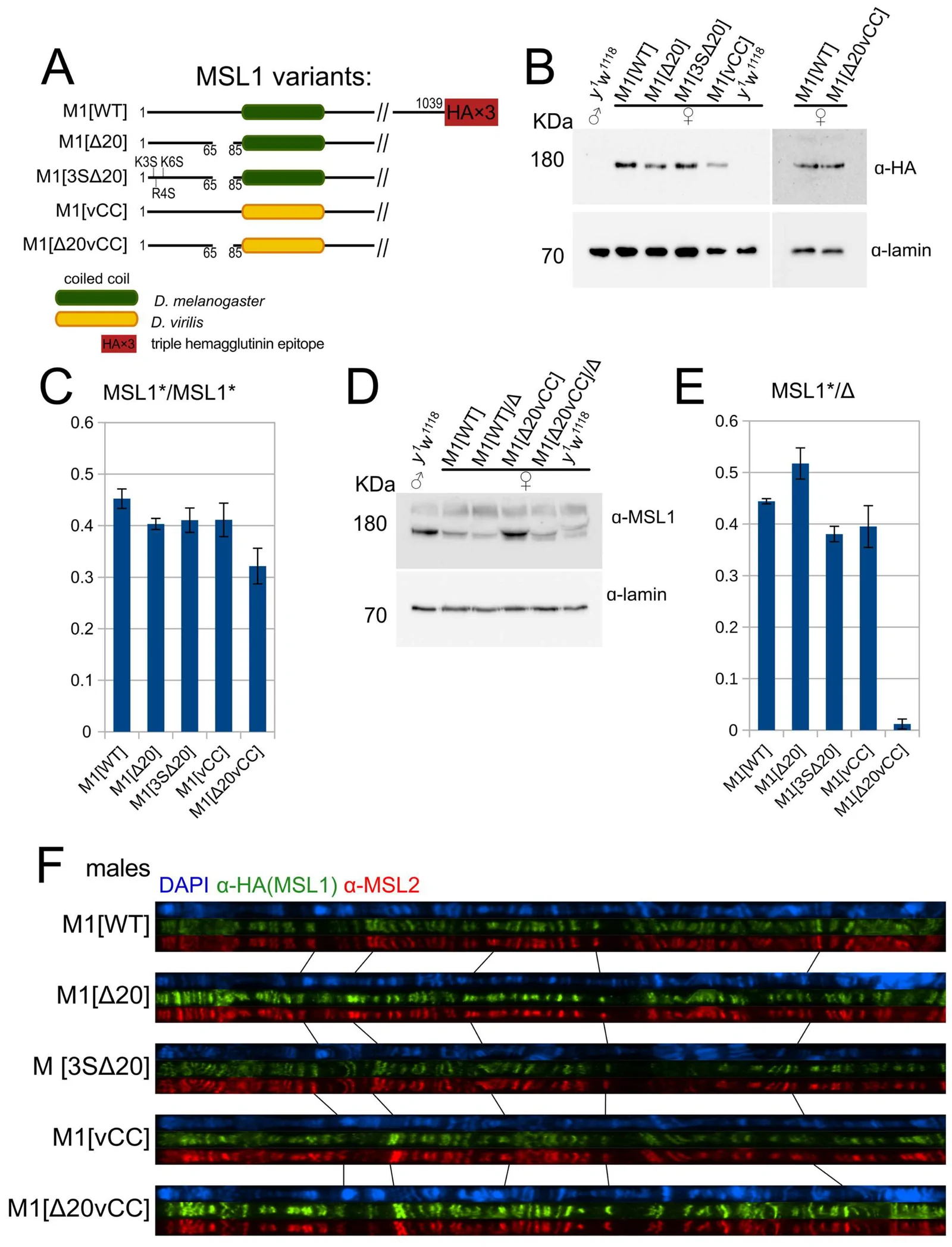
The role of the N-terminal region of MSL1 in dosage compensation. (**A**) A schematic of the MSL1 mutants generated. (**B**) Immunoblot analysis of protein extracts prepared from adult males (♂) and females (♀) of the lines *y^1^w^1118^* (control), M1[WT] (*msl-1^WT^*/*msl-1^WT^*), M1[Δ20] (*msl-1^Δ20^*/*msl-1^Δ20^*), M1[3SΔ20] (*msl-1^3SΔ20^*/*msl-1^3SΔ20^*), M1[vCC] (*msl-1^vCC^*/*msl-1^vCC^*), and M1[Δ20vCC] (*msl-1^Δ20vCC^*/*msl-1^Δ20vCC^*). Immunoblots were stained with anti-HA and anti-lamin Dm0 (internal control) antibodies. (**C**) The viability of males producing MSL1 mutants. The average proportion of msl-1*/msl-1* males in homozygous msl-1* lines. msl-1* denotes any transgene expressing one of the tested MSL1 variants. Error bars represent the standard error of the mean (SEM). Input data for the plots are presented in Table S2. (**D**) Immunoblot analysis of protein extracts prepared from 2-day males and females of the line *y^1^w^1118^* (control), and 2-day females of the lines M1[WT] (*msl-1^WT^*/*msl-1^WT^*), M1[WT]/Δ (*msl-1^WT^*/*msl-1^attP^*), M1[Δ20vCC] (*msl-1^Δ20vCC^*/*msl-1^Δ20vCC^*), and M1[Δ20vCC]/Δ (*msl-1^Δ20vCC^*/*msl-1^attP^*). Immunoblots were stained with anti-MSL1 and anti-lamin Dm0 (internal control) antibodies. (**E**) The viability of males in heterozygous (msl-1*/*msl-1^attP^)* F1 progeny from crosses between *msl-1^attP^*/*msl-1^attP^* females and msl-1*/msl-1* males. The average proportion of msl-1*/*msl-1^attP^* males in heterozygous (msl-1*/*msl-1^attP^)* progeny. msl-1* denotes any transgene expressing one of the tested MSL1 mutants. Error bars represent the standard error of the mean (SEM). Input data for the plots are presented in Table S2. (**F**) Cytological localization of MSL1 and MSL2 on polytene chromosomes obtained from the salivary glands of male larvae of the M1[WT], M1[Δ20], M1[3SΔ20], M1[vCC], and M1[Δ20vCC] lines.

Our results showed that the entire N-terminus of MSL1 is involved in diverse protein–protein interactions, but aa residues 1–15 and 66–85, as well as the coiled-coil domain, are the most important. Because we previously found that aa residues 1–15 and 41–65 are critical for interaction with *roX1* and *roX2* [23], here we focused on investigating the role of aa residues 66–85 and the coiled-coil domain of MSL1 in the specific recruitment of the MSL complex to HASs/CESs on the X chromosome in males. We generated a series of transgenic lines expressing MSL1 mutants fused at the C-terminus to a 3×HA epitope under the control of a previously employed promoter from the *Ubi-p63E* gene [23]. We integrated all transgenic constructs into the *msl-1^attP^* platform using a φC31-based integration system [60] (Figure S2), obtaining the following transgenic lines: control *msl-1^WT^* (wild-type MSL1), *msl-1^Δ20^* (deletion of aa residues 66–85 in MSL1), and *msl-1^vCC^* (replacement of the coiled-coil domain with the vCC domain in MSL1). Since the first 15 aa residues of the MSL1 protein are required for stability, we substituted three conserved basic aa residues—lysine 3, arginine 4, and lysine 6—with serines (K3S, R4S, and K6S, respectively; jointly referred to as 3S). In previous work [23], we found that expressing mutant MSL1^3S^ almost completely restored the viability of null *msl-1^−^* mutant males and maintained normal MSL complex binding to the X chromosome. Therefore, we created a transgenic line, *msl-1^3SΔ20^*, combining the 3S and Δ20 changes. Finally, to further weaken the interaction between MSL1 and C2H2 proteins, we generated a transgenic line expressing a mutant of MSL1 combining the vCC domain with the Δ20 deletion (MSL1^Δ20vCC^, Figure 3A). We were able to obtain and maintain all of these lines in a homozygous state, suggesting that these MSL1 mutants are at least partially functional.

To compare the abundance of MSL1 mutants produced by the transgenic lines, we measured their levels in 2–3-day-old homozygous females. Immunoblot analyses showed that all MSL1 mutants were produced in transgenic flies at nearly equivalent levels to MSL1^WT^ (Figure 3B). Thus, the modifications introduced into the N-terminal region did not affect the stability of the MSL1 mutants.

Because females homozygous for the *msl-1^attP^* allele remained viable, but males died during the larval stages [5,6], we examined the relative survival rate of homozygous males versus females. The *msl-1^WT^* line maintained an equal ratio between homozygous males and females, suggesting normal functioning of the MSL complex. The *msl-1^Δ20^*, *msl-1^3SΔ20^*, and *msl-1^vCC^* lines showed slightly lower proportion of males, likely reflecting minor disturbances in the functioning of the MSL complex (Figure 3C). Notably, for *msl-1^Δ20vCC^*, homozygous males hatched 5–6 days later than homozygous females. Thus, these results suggest that Δ20 deletion and the vCC domain have an additive effect in impairing MSL1 function.

Overexpression of MSL1 or MSL2 has been previously shown to partially compensate for inactivation of MSL complex components [23,61,62]. Because the MSL1 mutants are expressed at significantly higher levels than native MSL1 in *y^1^w^1118^* males, we also examined the viability of males that are heterozygous for one of the *msl-1* mutations and deletion of the *msl-1* gene (msl-1*/*msl-1^attP^*). In *msl-1^3SΔ20^*/*msl-1^attP^* heterozygotes, expression of MSL1 mutants was approximately twofold lower (Figure 3D). Again, males carrying *msl-1^WT^*, *msl-1^Δ20^*, *msl-1^3SΔ20^*, or *msl-1^vCC^* and *msl-1^attP^* exhibited nearly wild-type viability, while almost all males carrying *msl-1^Δ20vCC^*/*msl-1^attP^* died (Figure 3E). Thus, the simultaneous replacement of the coiled-coil domain with the vCC domain and the deletion of aa residues 66–85 of MSL1 have a synergistic effect in inactivating dosage compensation, which is most clearly observed in the heterozygous msl-1*/*msl-1^attP^* background.

We next examined the ability of the MSL1 mutants to bind the polytene chromosomes from larval salivary glands. Immunostaining of polytene chromosomes from the salivary glands of *Drosophila* larvae enables the visualization of proteins on interphase chromatin and has been used extensively to study dosage compensation [7,16,61,63,64,65]. In males of the *msl-1^WT^* line, MSL1 and MSL2 completely covered the polytene X chromosome. We obtained similar results with males of the *msl-1^Δ20^*, *msl-1^3SΔ20^*, *msl-1^vCC^*, and *msl-1^Δ20vCC^* lines, confirming that these MSL1 mutants retain functionality in the dosage compensation (Figure 3F and Figure S3). Unfortunately, we were unable to obtain polytene chromosome preparations from *msl-1^Δ20vCC^/msl-1^attP^* male larvae, which would likely have revealed abnormalities in MSL complex recruitment. These data indicate that the single mutants retain substantial MSL complex binding to the X chromosome and remain largely functional in dosage compensation. However, polytene chromosome staining is a low-resolution cytological assay and does not report transcriptional output; therefore, we cannot exclude subtle, site-specific differences in MSL complex binding or dosage compensation.

### 2.4. Simultaneous Deletion of aa Residues 66–85 and Replacement of the Coiled-Coil Domain with the vCC Domain in MSL1 Affects the Recruitment of the MSL Complex to the X Chromosome in Males

To further explore the role of the N-terminal region of MSL1 in the recruitment of the MSL complex to the X chromosome, we evaluated the binding of MSL1 and MSL2 by chromatin immunoprecipitation followed by next-generation sequencing (ChIP-seq) using chromatin prepared from 3-day-old adult *msl-1^WT^*, *msl-1^Δ20vCC^*, *msl-1^3SΔ20^*, and *msl-1^vCC^* males and a standard protocol for chromatin fragmentation using sonication, obtaining MSL1 and MSL2 profiles from two biological replicates.

**Figure 4:**
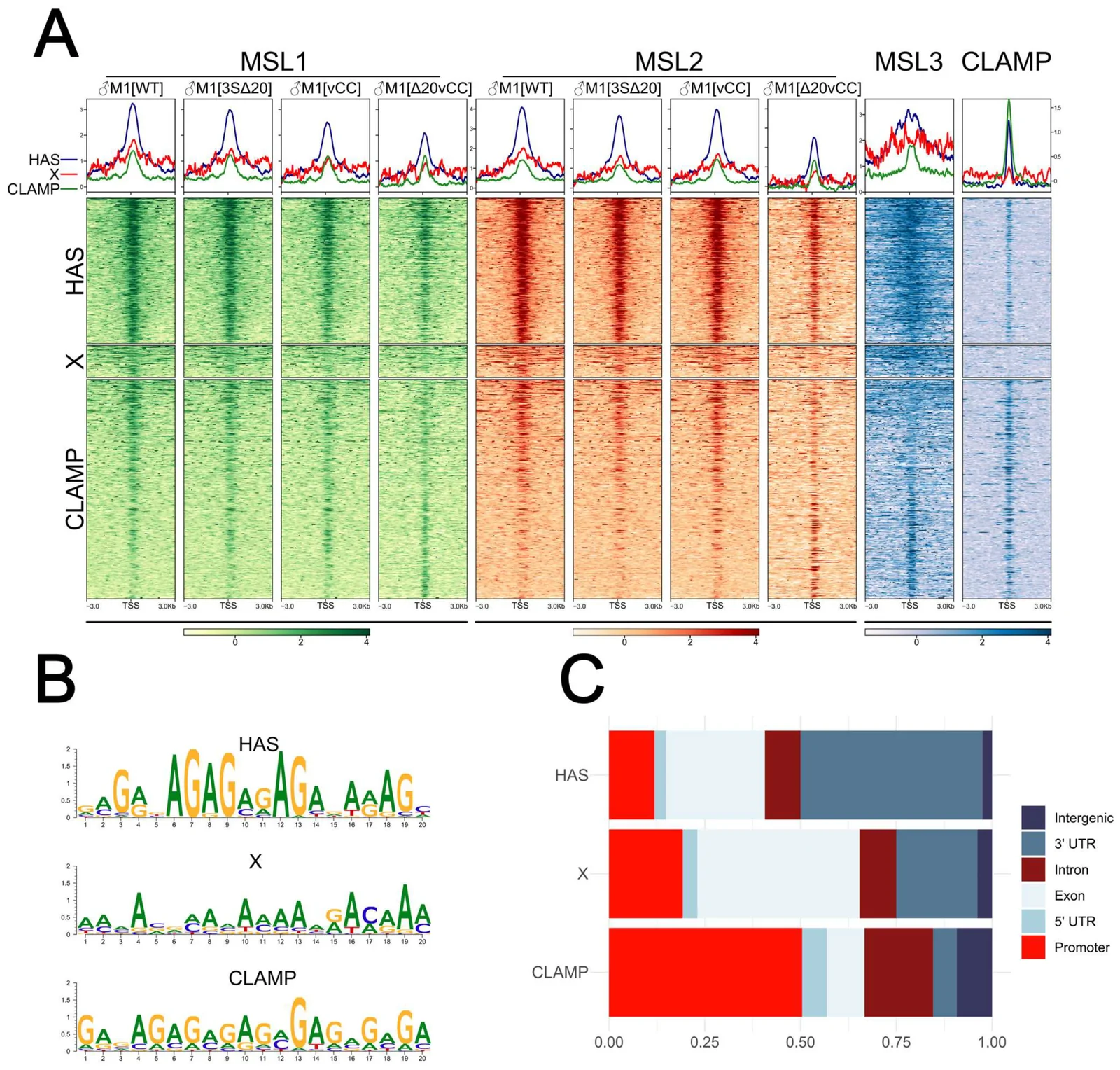
Comparisons of binding patterns of MSL mutants in adult males (♂)**.** (**A**) The average signal (top) and a heatmap (bottom) of the log fold-change (FC) between normalized (RPKM) test signals and nonspecific IgG signals for MSL1-binding sites in males of the lines M1[WT] (*msl-1^WT^*/*msl-1^WT^*), M1[3SΔ20] (*msl-1^3SΔ20^*/*msl-1^3SΔ20^*), M1[vCC] (*msl-1^vCC^*/*msl-1^vCC^*), and M1[Δ20vCC] (*msl-1^Δ20vCC^*/*msl-1^Δ20vCC^*). MSL1-binding sites are grouped into HAS/CES (HAS), broad peaks on the X chromosome (X), and CLAMP-rich peaks (CLAMP). (**B**) Motif logos for each group of MSL1 and MSL2 binding sites. (**C**) Genomic distribution of MSL1-binding sites in each group.

Unexpectedly, we found a complete correlation in the binding of MSL1 and MSL2 to genomic regions. The MSL1/MSL2 binding regions could be divided into three groups (Figure 4A and Figure S4). The core group (designated HAS) includes the previously characterized 236 HASs/CESs on the X chromosome that the MSL complex binds most efficiently [14,28,29,66]. Motif analysis using ChIPMunk revealed the expected GA-rich motif, or MRE, characteristic of HASs/CESs (Figure 4B). CLAMP binds to the core of most HASs/CESs and is believed to be a key DNA-binding protein in the recruitment of the MSL complex [34,35]. In adult *msl-1^WT^* males, MSL1 and MSL2 were closely associated with HASs/CESs, forming distinct peaks that coincided with the CLAMP binding site. Although MSL3 formed a peak in association with MSL1/MSL2, its binding also extended into surrounding regions. As previously shown [28,29], HASs/CESs have an unusual genomic distribution and are predominantly localized in the 3′ untranslated region (UTR) of actively transcribed genes (Figure 4C).

We also identified an additional group of MSL1/MSL2 binding sites on the X chromosome, designated X, that are characterized by broad peaks for MSL1 and MSL2 (Figure 4A and Figure S4). Such MSL1/MSL2 peaks also show enrichment for MSL3. The characteristic features of group X are the absence of GA-rich motifs and the absence of co-localization with CLAMP (Figure 4A,B and Figure S4). Like HAS/CES, the X sites are predominantly located in the body of actively transcribed housekeeping genes (Figure 4C and Figure S4). Interestingly, MSL1/MSL2 regions are typically located in close proximity to HAS/CES (Figure S4A) and may reflect the spread of the MSL complex across X-chromosome genes from HASs/CESs through an as-yet unknown mechanism.

The largest group of MSL1/MSL2 sites, designated CLAMP, is located on the X chromosome and autosomes and contains GA-rich motifs to which CLAMP binds (Figure 4A,B and Figure S4). The CLAMP sites also colocalize with MSL3, suggesting binding of the full MSL complex, and are predominantly located in the promoters of housekeeping genes (Figure 4C and Figure S4). MSL1/MSL2 binding to CLAMP sites is 2–3 times weaker than to HASs/CESs (Figure 4A and Figure S4), consistent with the efficient recruitment of the MSL complex only to HASs/CESs.

Consistent with the immunostaining results of polytene chromosomes in males, we observed no significant changes in MSL1 and MSL2-binding patterns to HASs/CESs in *msl-1^vCC^* or *msl-1^3SΔ20^* males compared with *msl-1^WT^* males (Figure 4A and Figure S4). These results confirmed that the vCC domain retains the ability to interact with MSL2 in vivo, consistent with the evolutionary conservation of the core functions of coiled-coil domains across long-diverged *Drosophila* species. At autosomal sites, MSL1^vCC^ and MSL1^3SΔ20^ binding was also comparable to that of MSL1^WT^. Altogether, these results suggest that inactivating individual domains in the N-terminal region of MSL1 has a minimal impact on the efficiency of MSL complex binding to the X chromosome and dosage compensation in males.

Next, we compared *msl-1^WT^* and *msl-1^Δ20vCC^* males, which have impaired dosage compensation. We observed a visible decrease in the binding of MSL1^Δ20vCC^ and MSL2 to HAS/CES (Figure 4A and Figure S4), as well as in the binding of MSL1^Δ20vCC^/MSL2 to the X sites. These results can be explained by MSL1^Δ20vCC^ being less efficient than MSL1^WT^ in recruiting the MSL complex to HASs/CESs in males.

### 2.5. The Coiled-Coil Domain of MSL1 Is Critical for MSL Complex Binding to the X Chromosome in Females Expressing MSL2

To further investigate the role of aa residues 66–85 and the coiled-coil domain of MSL1, we used the sensitive model system to examine factors influencing the specific recruitment of the MSL complex to the X chromosome. The MSL complex can assemble on the X chromosome in females expressing the *msl-2* gene [63,67,68,69,70]. In this sensitive model system, the MSL complex binds to the X chromosome less stably due to the relatively weak expression of *lncRNA:roX1* and *lncRNA:roX2* in females, which are required for efficient assembly of the MSL complex [62,71,72]. The female model ectopically expressing MSL2 is more sensitive to alterations in MSL1 function, which allows for the detection of subtle changes in MSL complex recruitment.

**Figure 5:**
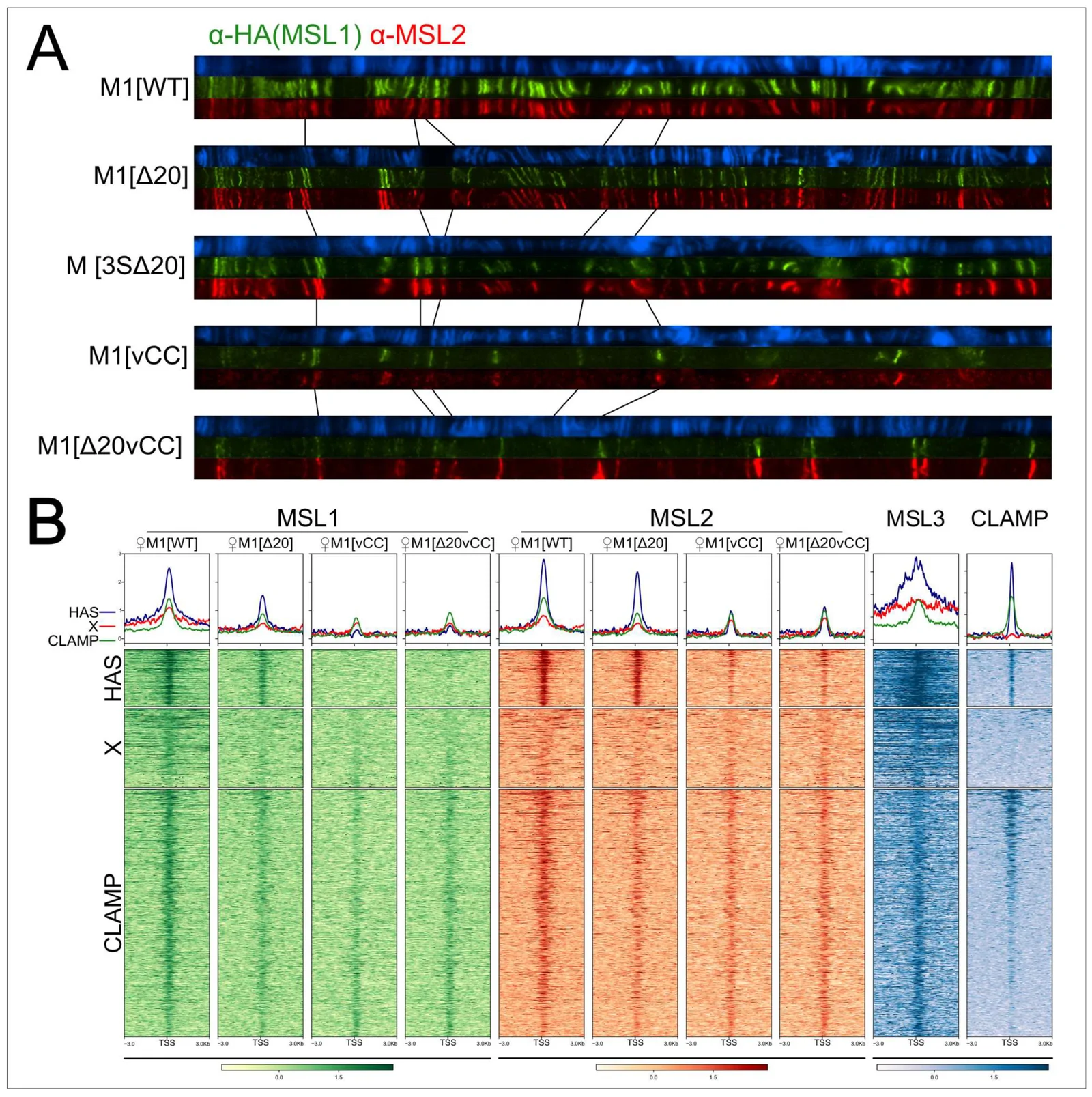
Comparisons of the binding patterns of MSL1 mutants in adult females (♀) ectopically expressing MSL2. (**A**) Cytological localization of MSL1 and MSL2 on polytene chromosomes obtained from the salivary glands of female larvae of the lines M1[WT]/M2 (*msl-1^WT^*/*msl-2^WT^*), M1[Δ20]/M2 (*msl-1^Δ20^*/*msl-2^WT^*), M1[3SΔ20]/M2 (*msl-1^3SΔ20^*/*msl-2^WT^*), M1[vCC]/M2 (*msl-1^vCC^*/*msl-2^WT^*), and M1[Δ20vCC]/M2 (*msl-1^Δ20vCC^*/*msl-2^WT^*). The panels show immunostaining using anti-HA (MSL1, green) and anti-MSL2 (red) antibodies, along with DAPI staining of polytene chromosome squashes (blue). Other designations are as in Figure 4. (**B**) The average signal (top) and a heatmap (bottom) of the log fold-change (FC) between normalized (RPKM) test signals and nonspecific IgG signals for MSL1-binding sites in females of the lines M1[WT]/M2, M1[Δ20]/M2, M1[vCC]/M2, and M1[Δ20vCC]/M2. MSL1-binding sites are grouped into HASs/CESs (HASs), broad peaks on the X chromosome (X), and CLAMP-rich peaks (CLAMP).

To establish a model system, we integrated the *Ubi:msl-2^WT^-FLAG* transgene into the *msl-1^attP^* platform using a φC31-based integration system [60]. To examine the co-recruitment of MSL1 mutants and MSL2 to the X chromosome, we used the immunostaining of polytene chromosomes from female larvae obtained by crossing *msl-2^WT^*/*CyO*, GFP males and msl-1*/msl-1* females. We examined the binding capabilities of the MSL2-FLAG and MSL1 variants on polytene chromosomes from the salivary glands of female msl-1*/*msl-2^WT^* larvae (Figure 5A and Figure S5). The *msl-1^3SΔ20^*/*msl-2* ^WT^ and *msl-1^Δ20^*/*msl-2^WT^* females showed similar modest reductions in MSL1 and MSL2 binding to the X chromosome compared with *msl-1^WT^*/*msl-2^WT^* females (Figure 5A and Figure S5). These results suggest that aa residues 66–85 are involved in the recruitment of the MSL complex to the X chromosome. The additional substitution of three conservative aa residues (3S) at the N-terminus of MSL1 did not further decrease the binding of the MSL complex to the X chromosome.

In addition, *msl-1^vCC^*/*msl-2^WT^* females demonstrated significantly reduced MSL complex binding: only individual MSL1+MSL2 bands remained on the polytene X chromosome. Interestingly, the remaining MSL1+MSL2 bands showed intense staining, suggesting that the MSL complex retains effective binding only to a subset of HAS/CES on the X chromosome in females expressing *msl-2*. Surprisingly, the binding pattern of the MSL complex was not altered on the polytene chromosomes of *msl-1^Δ20vCC^*/*msl-2^WT^* female larvae (Figure 5A and Figure S5) compared with *msl-1^vCC^*/*msl-2^WT^* female larvae. Thus, the coiled-coil domain of MSL1 is essential for the specific recruitment of the MSL complex to the X chromosome in polytene chromosomes of larval salivary glands.

To examine abnormalities in MSL complex recruitment to chromosomes in MSL1 mutants in more detail, we examined the binding of MSL1 and MSL2 by ChIP-seq using chromatin obtained from 2–3-day-old females (*msl-1^WT^*/*msl-2^WT^*, *msl-1^Δ20vCC^*/*msl-2^WT^*, *msl-1^Δ20^*/*msl-2^WT^*, and *msl-1^vCC^*/*msl-2^WT^*), obtaining MSL1 and MSL2 profiles from two biological replicates. For comparison, we performed ChIP-seq of MSL1 and MSL2 in *msl-1^WT^* males.

In females, MSL1^WT^/MSL2 binding sites could be divided into the same previously described groups of MSL1/MSL2 binding sites: HASs/CESs, X (broad MSL1/MSL2 peaks located near HASs/CESs), and CLAMP (Figure 5B and Figure S6). In *msl-1^Δ20^*/*msl-2^WT^* females, MSL1 and MSL2 bound less strongly to all genomic sites, confirming a role for aa residues 66–85 in the recruitment of the MSL complex to chromatin. Interestingly, *msl-1^vCC^*/*msl-2^WT^* and *msl-1^Δ20vCC^*/*msl-2^WT^* females exhibited significantly reduced MSL1 and MSL2 binding to the HAS/CES and X sites, while their binding to the CLAMP sites remained at the same level as in *msl-1^Δ20^*/*msl-2^WT^* females (Figure 5B and Figure S6). As a result, MSL1/MSL2 binds to CLAMP sites more strongly than to HASs/CESs. These results further support the important role of the species specificity of the coiled-coil domain of MSL1 in the recruitment of the MSL complex to HASs/CESs.

## 3. Discussion

This study examined the potential role of MSL1 in the specific recruitment of the MSL complex to the X chromosome in males. We found that the unstructured N-terminal region and the adjacent coiled-coil domain are involved in interactions with numerous C2H2 proteins.

Several transcription factors that interact with MSL1 are well-known transcriptional regulators. In *Drosophila*, Sna and Kr are transcriptional repressors [73] that occasionally function as transcriptional activators [74,75]. Odd and Ci are well-known, highly conserved DNA-binding transcription factors that regulate genes involved in various *Drosophila* developmental processes [76,77,78]. Z4 has been previously identified as a partner of the nonspecific-lethal (NSL) complex [79] that binds to housekeeping gene promoters [80,81] and is a component of the telomeric repeat binding factor 2 (TERF2/TRF2)–dependent promoter complex [82]. Kipf functions in the ovary as a recruiter of Rhino (Rhi), which mediates transcription of PIWI-interacting RNA (piRNA) precursors at transposon-rich heterochromatic loci [53]. However, other transcription factors identified as MSL1 interactors in our study have not yet been characterized.

Our findings suggest that the entire 93-aa N-terminal sequence likely represents a single unstructured region that forms 1–3 interactions with each C2H2 protein. However, it remains unknown whether the coiled-coil domain of MSL1 can simultaneously interact with MSL2 and C2H2 proteins. Moreover, because most of the identified C2H2 proteins remain poorly characterized, it remains unknown which, if any, cooperate with CLAMP to recruit the MSL complex to HASs/CESs.

Our results showed that the interaction between C2H2 proteins and the coiled-coil domain of MSL1, which is highly conserved among *Drosophila* orthologs, is not conserved in *D. virilis*. The lack of conservation between the N-terminal regions across *Drosophila* species may be a consequence of the rapid evolution of interactions between MSL1 and C2H2 proteins [83,84]. The mechanisms of MSL complex recruitment in *D. melanogaster* and *D. virilis* have been shown to differ significantly [22]. Unstructured regions have an advantage over structured regions, as there is no limit on mutations that increase the stability of the interaction with C2H2 proteins. Interestingly, the unstructured 93-aa N-terminal region of MSL1 combines overlapping regions that determine interactions with a large group of C2H2 proteins and, at the same time, highly specific interactions with *roX1* and *roX2*. Our study did not analyze the mechanisms of interaction between MSL1 and C2H2 proteins in detail. Therefore, further research is warranted to map and characterize the interacting regions of C2H2 proteins to better understand the mechanism that enables the N-terminal unstructured region of MSL1 to interact with them.

The generally accepted model suggests that, initially, MSL complexes bind to numerous HASs/CESs on the X chromosome in males [18,24,62,85]. Then, the MSL complexes spread from the HASs/CESs to active genes on the X chromosome through long-distance chromatin contacts formed by pre-existing chromosome architecture [86,87]. *roX1* and *roX2* guide MSL complex assembly and its spread from HASs/CESs into flanking chromatin [21,27,88,89]. CLAMP binds to MREs and is responsible for chromatin opening [35,39,90]. MSL2 contributes to the specific recognition of HASs/CESs through its direct binding to GAGA-rich motifs [32] and interaction with CLAMP [35,36,37]. Furthermore, CLAMP interacts with the unstructured C-terminal region of MLE [39].

Our study demonstrated that the MSL complex, including MSL1 and MSL2, bound HAS/CES most efficiently, which are predominantly located in the gene body and 3′ UTR. However, many additional CLAMP-binding sites on the X chromosome and autosomes also recruit MSL1 and MSL2, but much less efficiently. Most are located in the promoter and 5′ UTR, as is common for transcription factors that stimulate promoters.

Our study showed that the coiled-coil domain and, to a lesser extent, aa residues 66–85 of MSL1 promote the specific recruitment of the MSL complex to HASs/CESs. This effect was clearly demonstrated using a sensitive model system based on the formation of the MSL complex on the X chromosome in females ectopically expressing MSL2. Inactivation of the coiled-coil domain and deletion of aa residues 66–85 (Δ20) had an additive impairing effect on dosage compensation, resulting in a significant prolongation of the male developmental period. The synergy between Δ20 and vCC is best explained by the redundancy of C2H2-binding determinants in the N-terminal region of MSL1. Δ20 removes one contact module, whereas vCC removes the C2H2-binding surface of the coiled-coil domain without affecting MSL2 binding. When either module remains, sufficient C2H2 interactions may persist; when both are removed, MSL1 can no longer use C2H2 proteins to stabilize the MSL complex at many HASs/CESs. Consistent with the impaired dosage compensation, the MSL complex showed significantly reduced binding efficiency to HAS/CES in mutant adult males. Similarly, inactivation of MSL2’s CLAMP-interacting domain or CXC domain alone, both of which interact with the GA-rich motif in the MRE, only weakly affected dosage compensation; however, their simultaneous inactivation significantly reduced MSL complex binding to the X chromosome [36]. Thus, it is likely that multiple protein–protein interactions determine the specificity of MSL complex recruitment to the X chromosome.

**Figure 6:**
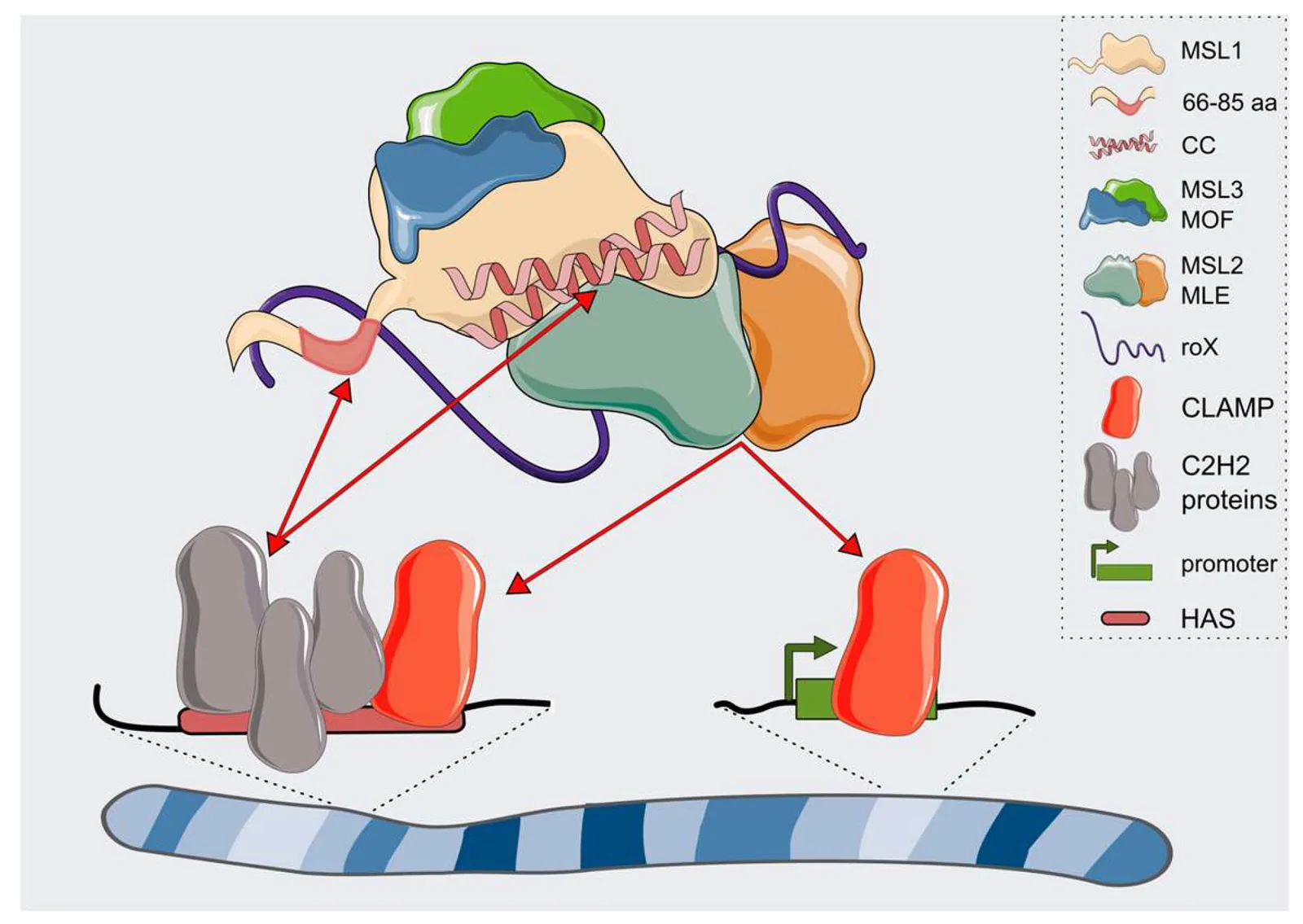
A hypothetical model of the specific recruitment of the MSL complex to HASs/CESs. A schematic of MSL complex (MSL1, MSL2, MSL3, MOF, MLE, and roX RNAs [roX1 and roX2]) recruitment to HASs/CESs (HASs) on the X chromosome. Presumably, additional C2H2 proteins are recruited to the HASs/CESs in cooperation with CLAMP. CLAMP interacts with MSL2 and MLE. C2H2 proteins interact with the coiled-coil domain (CC) and aa residues 66–85 of MSL1. Consequently, the MSL complex binds more stably to the HASs/CESs than to other regions at which the MSL complex interacts only with CLAMP.

We hypothesize that some C2H2 proteins that interact with the N-terminal region of MSL1 can functionally cooperate with CLAMP and bind specifically to HAS/CES (Figure 6). This hypothesis may explain why CLAMP binds to GA-rich motifs on all chromosomes but efficiently recruits the MSL complex only to HASs/CESs on the X chromosome of males. It is likely that MSL3 and MOF also contribute to the specific recruitment of the MSL complex by interacting with C2H2 proteins that bind to HASs/CESs. Further research is needed to confirm this hypothetical model.

## 4. Materials and Methods

### 4.1. Plasmid Construction

To express 3×FLAG-tagged C2H2 proteins and 3×HA-tagged MSL1[1–265] in the S2 cells, protein-coding sequences were cloned in frame with 3×FLAG and 3×HA, excised, and subcloned into the pAc5.1 plasmid (Life Technologies, Carlsbad, CA, USA).

Plasmids for the yeast two-hybrid assay were prepared using the full-sized and truncated versions with separate domains of MSL1 and C2H2 proteins as C-termini fused with pGBT9 and pGAD424 vectors from Clontech (Mountain View, CA, USA). PCR directed mutagenesis was used to make constructs encoding deletion variants of *msl-1* (corresponding primers are given in Table S3).

Different full-sized variants of *msl-1* were cloned into an expression vector containing *attB* site for φC31-mediated recombination [60], *Ubi-p63E* promoter with its 5′UTR, 3′UTR with *SV40* polyadenylation signal, *white* gene as a reporter for the detection of transformants (Figure S2).

### 4.2. Yeast Two-Hybrid Assay

Yeast two-hybrid assays were carried out using yeast strain pJ69-4A (*MATa trp1-901 leu2-3*, *112 ura3-52 his3-200 gal4Δ gal80Δ GAL2-ADE2 LYS2::GAL1-HIS3 met2::GAL7-lacZ*), with plasmid vectors from Clontech. In brief, for growth assays, AD (activation domain of GAL4)- and BD (DNA-binding domain of GAL4)-fused plasmids were co-transformed into yeast strain pJ69-4A by the lithium acetate method based on Clontech protocols. Transformed cells were plated on selective medium lacking Leu (leucine biosynthesis gene *LEU2* is expressed from pGAD424 plasmid) and Trp (tryptophan biosynthesis gene *TRP1* is expressed from pGBT9 plasmid) (SD-2). The plates were incubated at 30 °C for 2–3 days. Afterward, the colonies were streaked out on plates on second selective medium lacking either Leu, Trp and His (SD-3), or lacking adenine in addition to the three amino acids (SD-4), or lacking the three amino acids but containing 5 mM 3-amino-1,2,4-triazole (SD-3 + 5mM 3AT). The plates were incubated at 30 °C for 3–4 days, and growth was assessed. The positive growth of yeast on second selective medium indicates a physical interaction between tested protein molecules. Each assay was prepared as at least three independent biological replicates with three technical repeats.

### 4.3. Fly Crosses and Transgenic Lines

*Drosophila* lines were grown at 25 °C and 75% humidity under standard culture conditions. The transgenic constructs were injected into preblastoderm embryos using the φC31-mediated site-specific integration system [60] at *msl-1^attP^* line (Figure S2).

To derived females that expressed *msl-2* were obtained transgenic line that carrying *Ubi:msl-2^WT^-FLAG* transgene expressing the *msl-2* cDNA under the control of the *Ubi-p63E* promoter at the *msl-1^attP^* site and balanced by *CyO*, *GFP*.

To assess role of MSL1 variants in binding of the MSL complex to polytene chromosomes, virgin Ubi:msl-1*/Ubi:msl-1* females were crossed with *Ubi:msl-2^WT^-FLAG*/*CyO*, *GFP* males.

For further study, polytene chromosomes from the salivary glands of Ubi:msl-1*/*Ubi:msl-2^WT^-FLAG* larvae (males or females) were used to prepare preparations. The Ubi:msl-1*/*Ubi:msl-2^WT^-FLAG* females were also selected for Western blot analyses.

### 4.4. Antibodies

Antibodies against MSL2[421–540], MSL1[423–1030] were raised in rabbits and purified from the sera by ammonium sulfate fractionation followed by affinity purification on CNBr-activated Sepharose (GE Healthcare, Chicago, IL, USA) or Aminolink Resin (ThermoFisher Scientific, Waltham, MA, USA) according to standard protocols. Mouse monoclonal anti-HA (66006-2-lg, Proteintech, Rosemont, IL, USA) at a dilution of 1:500; mouse monoclonal anti-lamin Dm0, clone ADL84.12 (#ADL84.12, DSHB, Iowa City, IA, USA) at a dilution of 1:500.

### 4.5. Fly Extract Preparation

Ten adult flies were cooled in liquid nitrogen, homogenized with a Dounce homogenizer (10–20 times) in 500 µL of cold buffer A (15 mM HEPES pH 7.6, 60 mM KCl, 15 mM NaCl, 13 mM EDTA, 0.1 mM EGTA, 0.15 mM spermine, 0.5 mM spermidine, 0.5% NP-40, 0.5 mM DTT, 0.5 mM PMSF, 1:500 Halt™ Protease Inhibitor Cocktail, ThermoFisher Scientific), filtered through a 70 µm cell strainer and centrifuged at 800× *g* for 5 min at 4 °C. The supernatant was discarded, and the pellet was resuspended in 500 µL of cold nuclear lysis buffer (15 mM HEPES pH 7.6, 140 mM NaCl, 1 mM EDTA, 0.1 mM EGTA, 1% Triton X-100, 0.1% Sodium deoxycholate, 0.5 mM PMSF, 1:500 Halt™ Protease Inhibitor Cocktail), incubated on ice for 10 min and sonicated (2 times, 20 s on–60 s off, low power) using Bioruptor^®^ Plus (Diagenode, Denville, NJ, USA). The suspension was centrifuged at 3000× *g* for 10 min at 4 °C, and the supernatant was discarded. The pellet was washed 3 times with cold nuclear lysis buffer and then resuspended in 50 µL of nuclear lysis buffer. Laemmli buffer and urea (up to 2 M) were added, and the sample was incubated for 5 min at 95 °C and loaded on an SDS-PAGE gel.

### 4.6. Immunostaining of Polytene Chromosomes

*Drosophila* 3rd-instar larvae were cultured at 25 °C under standard conditions. Polytene chromosome staining was performed as described [91]. The following primary antibodies were used: rabbit anti-MSL2 at 1:500 dilution, mouse anti-HA at 1:500 dilution. The secondary antibodies were Alexa Fluor 488 goat anti-mouse 1:1000 and Alexa Fluor 555 goat anti-rabbit 1:1000 (Invitrogen, Carlsbad, CA, USA). The polytene chromosomes were prepared with mounting medium that include DAPI (LumiMount DAPI, Lumiprobe Rus, Moscow, Russian ). Images were acquired on the Nikon Eclipse T*i* fluorescent microscope using Nikon DS-Qi2 digital camera (Nikon Instruments, Melville, NY, USA), processed with NIS-Elements Viewer 5.21 (Nikon Instruments) and ImageJ 1.54p (Fiji distribution, https://fiji.sc/ accessed on 1 September 2026). A total of 3–4 independent staining and 4–5 samples of polytene chromosomes were performed with each MSL1-expressing transgenic line.

### 4.7. Co-Immunoprecipitation Assay

*Drosophila* S2 cells were grown in SFX medium (HyClone, Logan, UT, USA) at 25 °C. S2 cells grown in SFX medium were co-transfected by plasmid expressing 3×HA-MSL1[1–265] and plasmid expressing either of 3×FLAG-C2H2 or 3×FLAG-MSL2 with MACSFectin (Miltenyi Biotec, Gaithersburg, MD, USA), as recommended by the manufacturer. After transfection, the cells were incubated for 48 h, washed once with cold 1×PBS, and resuspended in 20 packed cell volumes of hypotonic lysis buffer (20 mM Tris-HCl, pH 7.4, with 10 mM KCl, 10 mM MgCl2, 2 mM EDTA, 10% glycerol, 1% Triton X-100, 1 mM DTT, 0.5 mM PMSF and Halt Protease Inhibitor Cocktail). After incubation on ice for 10 min, the cells were sonicated (2 × 15 s on ice at 20% output, 40% duty cycle), NaCl was added to a final concentration of 150 mM, and incubation on ice continued for 60 min, with periodic mixing. Sonication was repeated as above to reduce viscosity, cell debris was pelleted by centrifugation at 10,000× *g* for 30 min at 4 °C, and the supernatant was collected for immunoprecipitation with anti-HA and mouse IgG magnetic beads equilibrated in incubation buffer-150 (20 mM Tris-HCl, pH 7.4, with 150 mM NaCl, 10 mM MgCl2, 1 mM EDTA, 1 mM EGTA, 10% glycerol, and 0.1% NP-40). The protein extract (50 μg protein) was adjusted to a volume of 200 µL with buffer-150, mixed with magnetic beads (15 μL), and incubated on a rotary shaker overnight at 4 °C. The beads were then washed with five portions of buffer-150, resuspended in SDS-PAGE loading buffer, boiled, and analyzed by Western blotting. Proteins were detected using the Crescendo or Forte Western blotting substrate (Millipore, Burlington, MA, USA) with anti-FLAG-HRP antibodies (Sigma, St. Louis, MO, USA).

### 4.8. ChIP-seq

Samples of 500 mg each of adult flies were ground in a mortar in liquid nitrogen and resuspended in 10 mL of buffer A (15 mM HEPES-KOH, pH 7.6, 60 mM KCl, 15 mM NaCl, 13 mM EDTA, 0.1 mM EGTA, 0.15 mM spermine, 0.5 mM spermidine, 0.5% NP-40, 0.5 mM DTT) supplemented with 0.5 mM PMSF and Complete Protease Inhibitor Cocktail V (Calbiochem, San Diego, CA, USA). The suspension was then homogenized in a Potter (10 strokes) and subsequently in a Dounce Homogenizer (tight pestle, 15 strokes) homogenizers and filtered through 70 µm MACS SmartStrainers (Miltenyi Biotec). The homogenate was cross-linked with 1% formaldehyde for 15 min at room temperature. Cross-linking was stopped by adding glycine to a final concentration of 125 mM. The nuclei were washed with three 10 mL portions of wash buffer (15 mM HEPES-KOH, pH 7.6, 60 mM KCl, 15 mM NaCl, 1 mM EDTA, 0.1 mM EGTA, 0.1% NP-40, protease inhibitors) and resuspended in 1 mL of nuclear lysis buffer (15 mM HEPES, pH 7.6, 140 mM NaCl, 1 mM EDTA, 0.1 mM EGTA, 1% Triton X-100, 0.1% sodium deoxycholate, 0.5% N-lauroylsarcosine, 0.1% SDS, 0.5 mM PMSF, Calbiochem Complete Protease Inhibitor Cocktail V). The suspension was incubated for 30 min at 4 °C and then sonicated with Covaris ME220 (40 × 15 s with 30 s intervals at 6 °C, peak power 75.0, duty % factor 25, cycles/burst 1000), and 50 µL aliquots were used to test the extent of sonication and to measure DNA concentration. Chromatin was then transferred to 1.5 mL tubes and centrifuged at 14,000 rpm for 10 min at 4 °C. Corresponding antibodies (anti-Msl1, 1:300; anti-Msl2, 1:200; nonspecific rabbit IgG) were incubated for 1 h at room temperature with 20 µL aliquots of Protein A Dynabeads (Invitrogen) mixed with 200 µL of PBST. Then antibody–Dynabeads complexes were washed and equilibrated in nuclear lysis buffer. Chromatin samples containing 10–20 µg of DNA equivalent in 200 µL nuclear lysis buffer (2 µL aliquots of chromatin as input material) were incubated overnight at 4 °C with antibody–Dynabeads complexes. After three rounds of washing with nuclei lysis buffer supplemented with 500 mM NaCl, and TE buffer (10 mM Tris-HCl, pH 8; 1 mM EDTA), the DNA was eluted with elution buffer (50 mM Tris-HCl, pH 8.0; 1 mM EDTA, 1% SDS), treated with RNase A for 30 min and proteinase K overnight, incubated at 65 °C for 6 h and extracted with ChIP DNA Clean&Concentrator (Zymo Research, Irvine, CA, USA). At least two independent biological replicates were made for each chromatin sample.

The ChIP-seq libraries were prepared with NEBNext^®^ Ultra™ II DNA Library Prep kit (New England Biolabs, Ipswich, MA, USA), as described in the manufacturer’s instructions. PCR amplification was performed using NEB primers for 15–16 cycles using the Q5 Hot Start HiFi PCR Master Mix (New England Biolabs). The PCR-amplified library was purified using MagSelect beads (Magen Biotechnology, Guangzhou, China) and its quality was assessed on a Bioanalyzer 2100 system (Agilent Technologies, Santa Clara, CA, USA). Diluted libraries were clustered on a pair-read flowcell and sequenced using a NovaSeq 6000 system (Illumina, San Diego, CA, USA).

### 4.9. ChIP-seq Data Processing and Sequence Analysis

ChIP-seq data for MSL1, MSL2, and CLAMP were generated as two biological replicates using paired-end sequencing. MSL3 raw data were obtained from our previous work [23]. Read trimming and alignment were performed using cutadapt [92] Bowtie2 [93] following previously established protocols [49]. The dm6 assembly of the *Drosophila melanogaster* genome served as the reference. Biological replicates were merged using the samtools merge function [94]. Coverage tracks in bigWig format were generated with deepTools bamCoverage [95], and log2 fold-changes (log2FC) between protein and IgG control signals were calculated using deepTools bigwigCompare.

Peak calling was conducted only for MSL1 variants for males (MSL1^WT^, MSL1^vCC^, MSL1^3SΔ20^, and MSL1^Δ20vCC^) and females (MSL1^WT^, MSL1^Δ20^, MSL1^vCC^, and MSL1^Δ20vCC^) as described earlier [49].

1. PCR duplicates were removed using Picard tools (http://broadinstitute.github.io/picard/ (accessed on 1 September 2026)).
2. Blacklist regions were filtered out.
3. Peaks were called with MACS2 [96] against nonspecific IgG controls using a relaxed *p*-value threshold of 1 × 10^−2^ to enable subsequent irreproducible discovery rate (IDR) analysis [97] (https://github.com/nboley/idr (accessed on 1 September 2026)).

Reproducibility was evaluated via the IDR pipeline, applying *p*-value cutoffs of 0.05 for true replicates and 0.01 for pseudoreplicates. All samples showed good reproducibility, with a rescue ratio (RR) and self-consistency ratio (SR) below 2. Consequently, an optimal, highly reproducible peak set was selected for each sample.

Subsequent analyses were conducted in R version 4.1.3 (R Core Team (2023). R: A language and environment for statistical computing. R Foundation for Statistical Computing, Vienna, Austria. https://www.R-project.org/ accessed on 1 September 2026). To create a unified MSL1 peak list, the individual MSL1 peak lists were standardized to ±150 bp around summit positions and merged using the GenomicRanges package’s reduce function [98]. This consolidated peak list was then categorized into three genomic groups:

HAS: MSL1 peaks overlapping with unified High-Affinity Site (HAS) variants compiled from three studies published earlier [28,29,99].

X: MSL1 peaks on the X chromosome that do not overlap HASs and have CLAMP signal ≤ 0.6 logFC versus control.

CLAMP: MSL1 peaks that do not overlap HASs and have CLAMP signal > 0.6 logFC.

Genomic annotations for the unified MSL1 peaks were assigned with the annotatePeak function from the ChIPseeker package version 1.32.0 [100].

Motif discovery across these five groups was performed using ChIPMunk v8 [101]. Heatmaps and signal profiles were generated with the deepTools plotHeatmap function.

## 5. Conclusions

In this study, we demonstrated that the unstructured N-terminal region and the adjacent coiled-coil domain of MSL1 are actively involved in interactions with numerous C2H2 proteins. Although four representative interactions were confirmed by co-immunoprecipitation, the remaining Y2H-positive interactions should be regarded as candidates for further validation. A comprehensive functional analysis of individual C2H2 proteins and their roles in dosage compensation will be the subject of a separate study.

While the coiled-coil domain is highly conserved among *Drosophila* species, its interaction with C2H2 proteins is species-specific and is not conserved in *Drosophila virilis*. Furthermore, we found that the coiled-coil domain and amino acid residues 66–85 of the MSL1 protein are critical for the specific recruitment of the MSL complex to high-affinity sites (HASs/CESs) on the male X chromosome. Based on these findings, we propose a model in which specific C2H2 proteins interacting with the N-terminal region of MSL1 functionally cooperate with CLAMP, thereby driving the highly specific and stable recruitment of the entire MSL complex to HASs/CESs on the X chromosome. Direct demonstration of simultaneous binding of C2H2 proteins with the MSL complex and HASs/CESs requires further experiments.

Although the *Drosophila* MSL complex and mammalian dosage compensation operate through fundamentally different strategies, both rely on lncRNA-mediated recruitment of chromatin-modifying activities and on combinatorial interactions between DNA-binding architectural proteins. The principles uncovered here—particularly the use of an unstructured N-terminal region and a coiled-coil domain to integrate multiple C2H2 protein inputs—may help guide future studies of mammalian MSL1/NSL complexes and other chromatin regulatory systems.

## Data Availability

Requests for further information and resources should be directed to and will be fulfilled by the lead contacts, Oksana Maksimenko (maksog@mail.ru). Strains are available from the corresponding author upon request. The ChIP-seq data are available on GEO NCBI under the accession number GSE309026.

## Supplementary Materials

The following supporting information can be downloaded at: https://www.mdpi.com/article/10.3390/ijms27219861/s1.

## Abbreviations

The following abbreviations are used in this manuscript:

aa	amino acid
C2H2	C2H2-type zinc finger
CC	coiled-coil
CES	chromatin entry site
ChIP-seq	chromatin immunoprecipitation followed by next-generation sequencing
CLAMP	chromatin-linked adaptor for MSL proteins
HAS	high-affinity site
MLE	maleless
MRE	MSL recognition element
MSL	male-specific lethal
PionX	pioneering sites on the X
RING	really interesting new gene
roX	RNA on the X
vCC	coiled-coil domain of Drosophila virilis MSL1
Y2H	Yeast two-hybrid
