# Supplementary material for: The N-Terminal Region of Drosophila MSL1 Interacts with Zinc Finger Proteins and Promotes the Recruitment of the MSL Complex to the X Chromosome in Males
Source: Int J Mol Sci. 2026 Nov 3;27(21):9861. doi: 10.3390/ijms27219861 (PMC13764204; doi:10.3390/ijms27219861)
Supplement: Supplementary file 1 [file ijms-27-09861-s001.zip › Figures S1-S6.pdf]

# Supplementary Figures

## The N-terminal region of *Drosophila* MSL1 interacts with zinc finger proteins and promotes the recruitment of the MSL complex to the X chromosome in males

Valentin Babosha, Varvara Ryzhkova, Anastasia Revel-Muroz, Evgeniya Tikhonova, Yulia Vasileva, Oksana Maksimenko, Pavel Georgiev

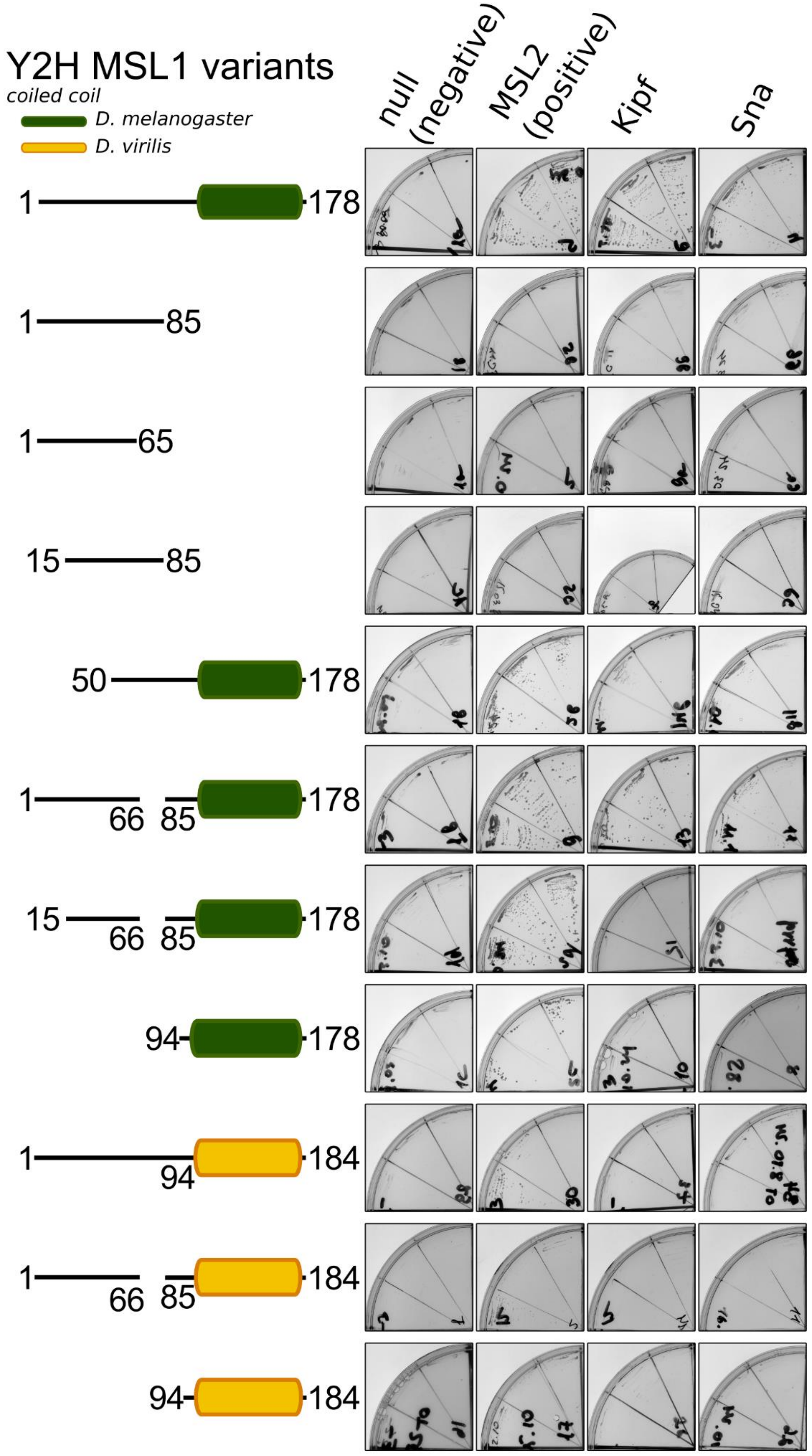

Y2H MSL1 variants
coiled coil
D. melanogaster
D. virilis
null (negative)
MSL2 (positive)
Kipf
Sna
1
178
1
85
1
65
15
85
50
178
1
66
85
178
15
66
85
178
94
178
1
94
184
1
66
85
184
94
184

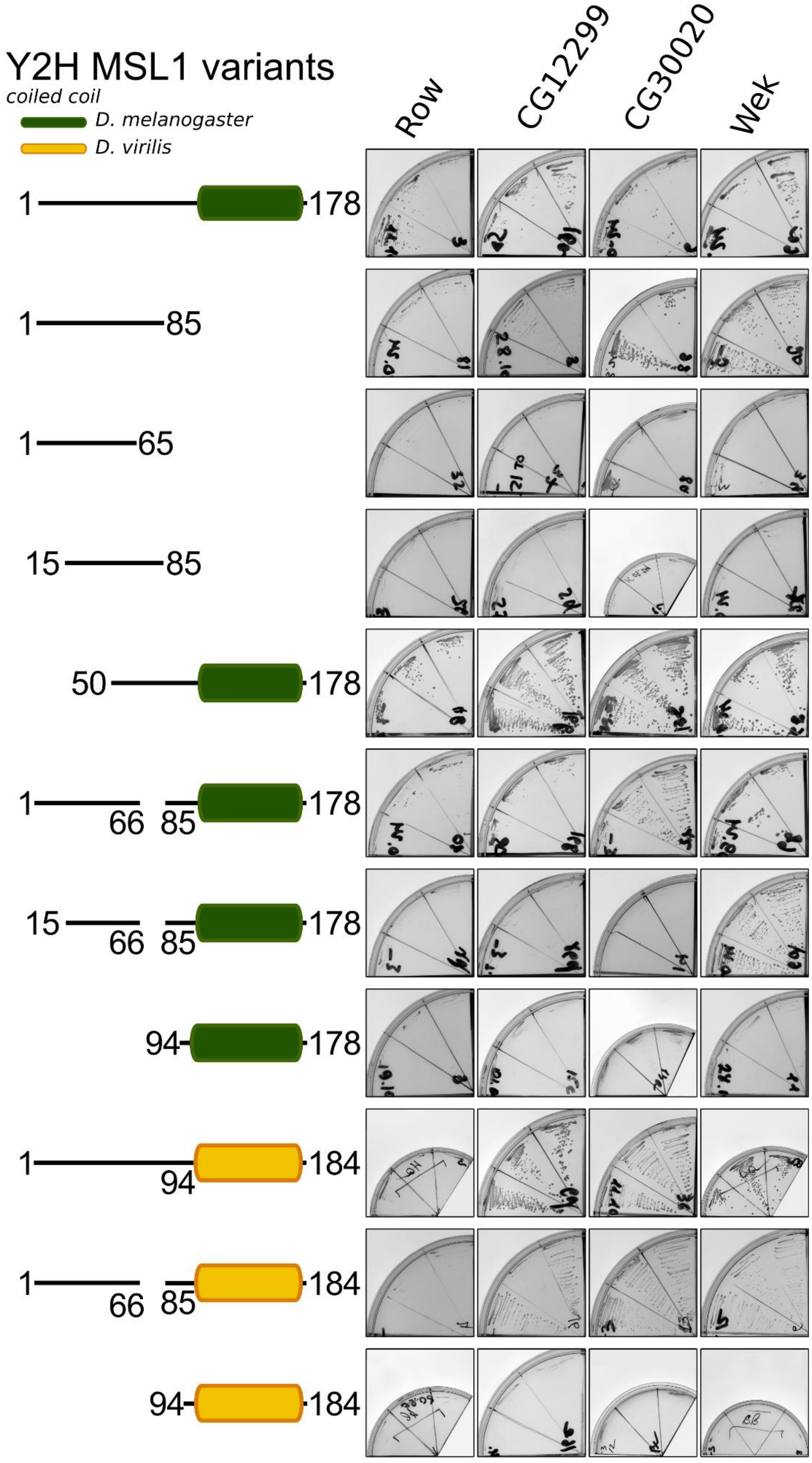

Y2H MSL1 variants
coiled coil
D. melanogaster
D. virilis
Row
CG12299
CG30020
Wek
1
178
1
85
1
65
15
85
50
178
1
66
85
178
15
66
85
178
94
178
1
94
184
1
66
85
184
94
184

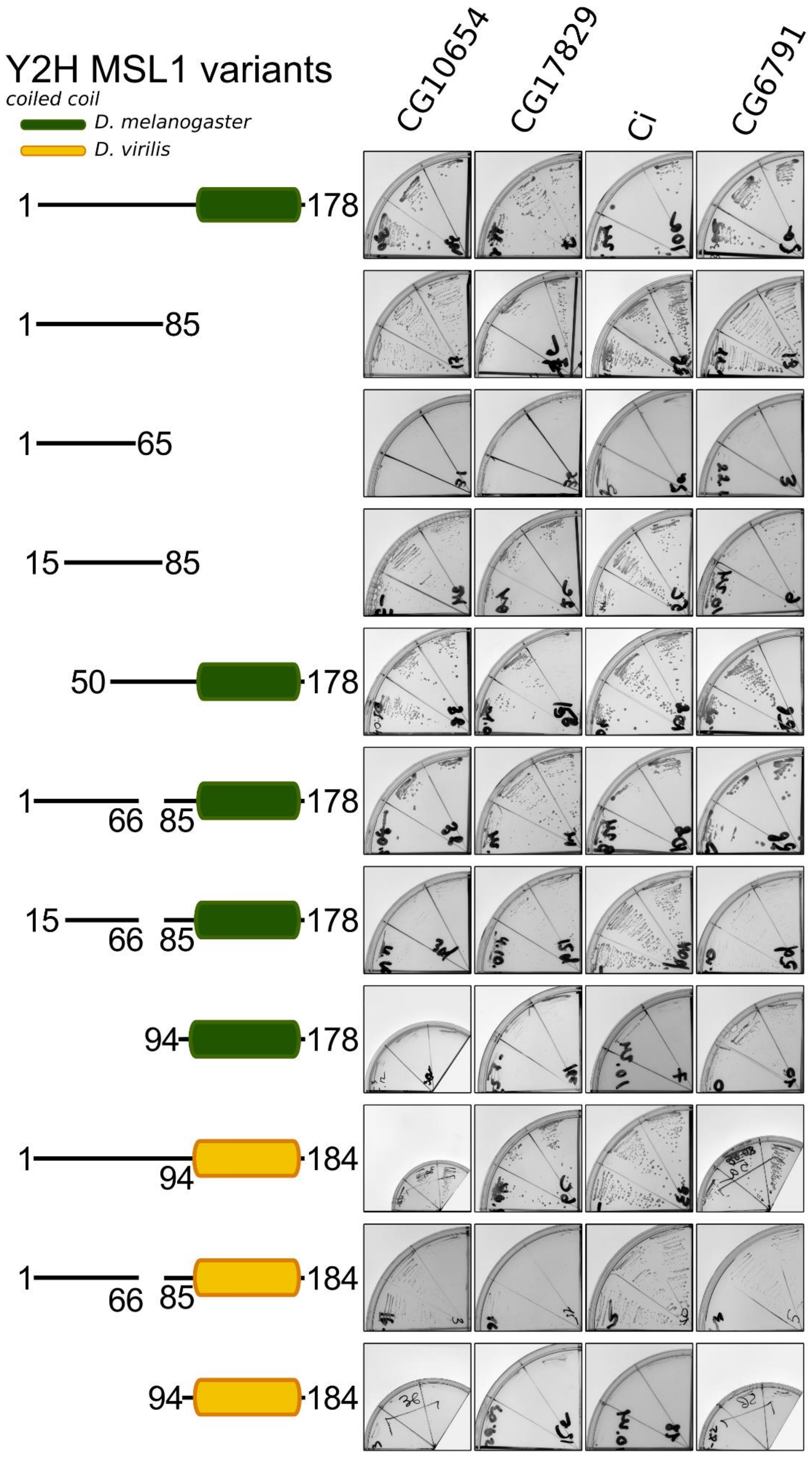

Y2H MSL1 variants
coiled coil
D. melanogaster
D. virilis
CG10654
CG17829
Ci
CG6791
1
178
1
85
1
65
15
85
50
178
1
66
85
178
15
66
85
178
94
178
1
94
184
1
66
85
184
94
184

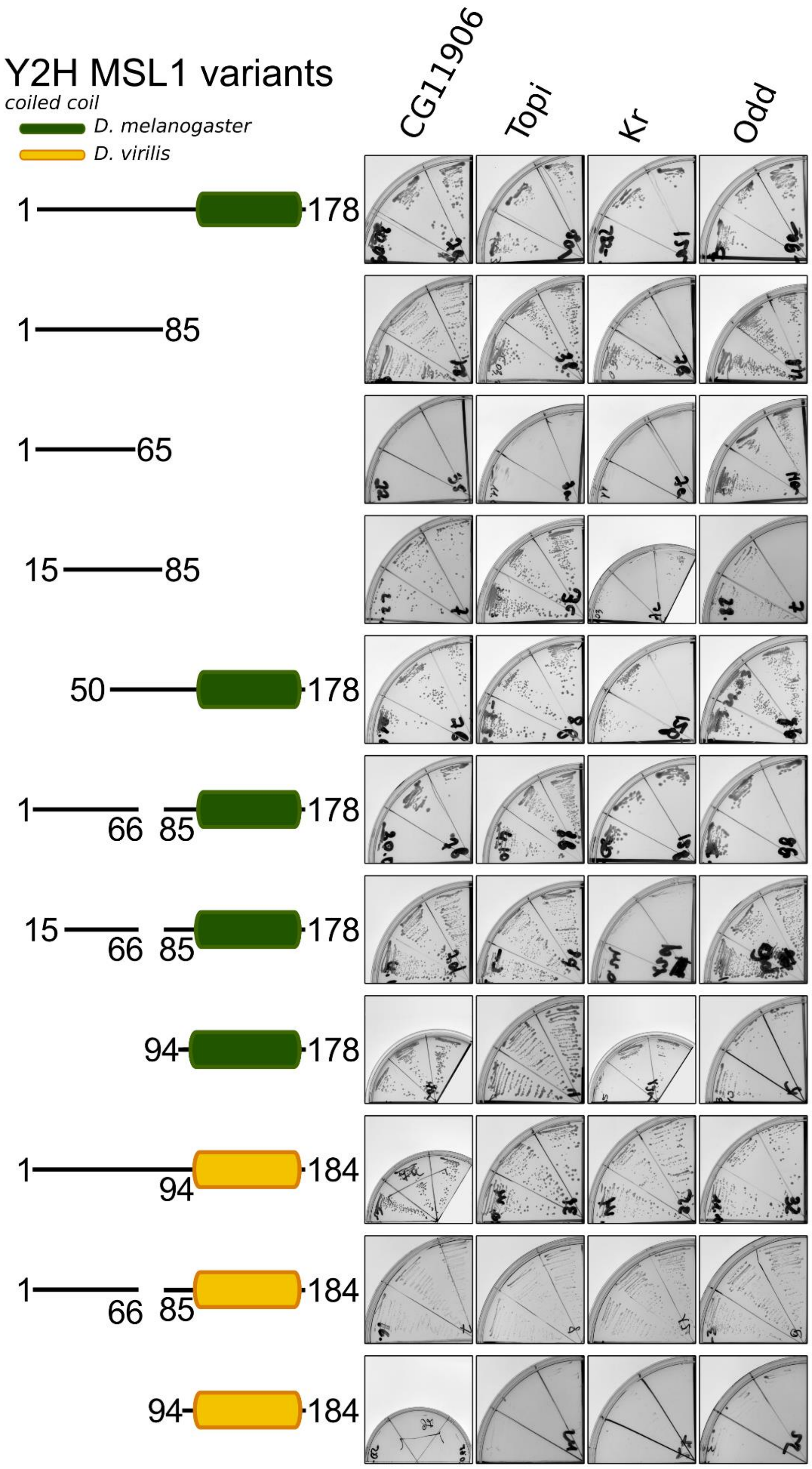


**Fig. S1. Mapping regions in MSL1[1–178] that interact with 14 C2H2 proteins in the Y2H assay.** MSL1[1–178] (truncated MSL1 containing only aa residues 1–178) mutants were fused to the GAL4 DNA-binding domain and tested for interaction with MSL2 (positive control) or C2H2 proteins fused to the GAL4 activating domain. Growth assay plates without tryptophan, leucine, and histidine are shown (yeast are unable to grow on this medium in the absence of an interaction).

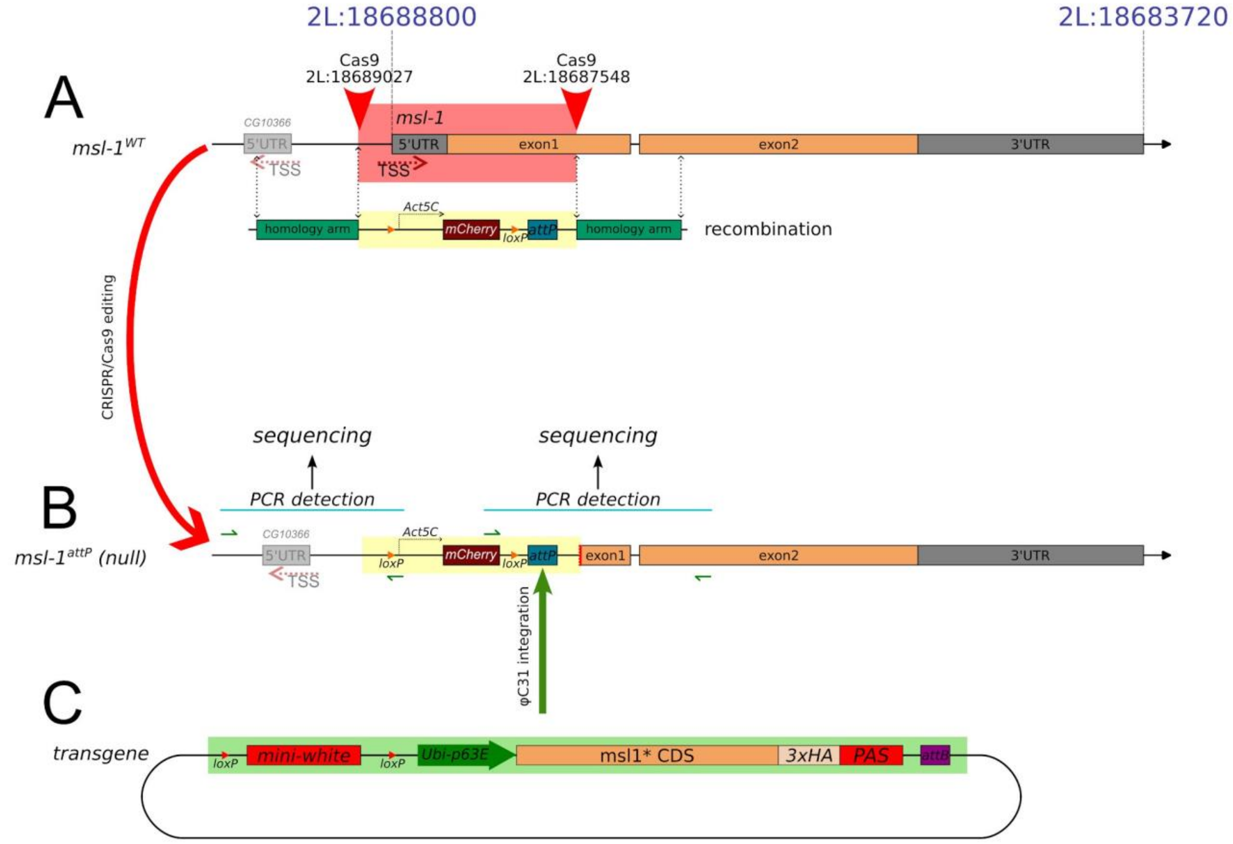


**Fig. S2. CRISPR/Cas9 editing of the *msl-1* gene. (A)** The *msl-1* gene and the recombination matrix. Coordinates of the gene, CRISPR targets, and homology arms are shown, along with the gene parts, reporters, recombination sites, and primers. **(B)** The resulting allele *msl-1*$^{attP}$ included the attP recombination site and the reporter gene *mCherry*. Successful CRISPR/Cas9 editing was confirmed by detecting both PCR products from two primer pairs (msl1-us5_F/5'lx-pAc_R and 3'mCh_D/msl1-ds3_R). These were then validated by Sanger sequencing. **(C)** The scheme of the transgenic construct, which was then integrated into the recombination site of the resulting allele, *msl-1*$^{attP}$. "msl1* CDS" denotes the coding sequences of MSL1$^{WT}$, MSL1$^{\Delta 20}$, MSL1$^{m3S\Delta 20}$, MSL1$^{vCC}$, and MSL1$^{\Delta 20vCC}$.

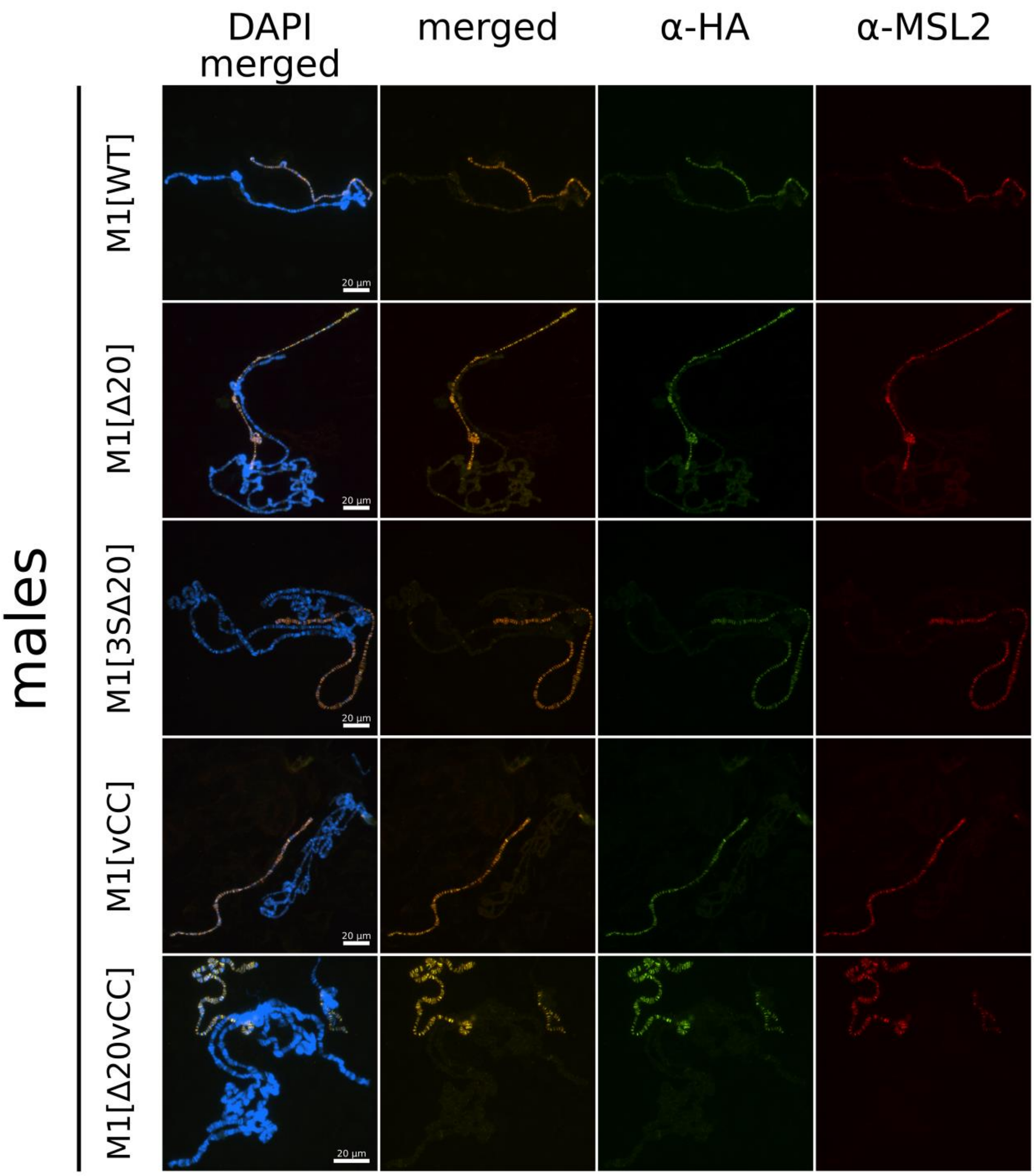


**Fig. S3. Distribution of the MSL complex on the polytene chromosomes from third instar male larvae expressing both MSL1* mutants.** The panels show immunostaining with mouse anti-HA antibody (green) and rat anti-MSL2 antibody (red), along with DAPI staining of DNA (blue). The following lines were examined: M1[WT] (*msl-1*$^{WT}$/*msl-1*$^{WT}$), M1[Δ20] (*msl-1*$^{Δ20}$/*msl-1*$^{Δ20}$), M1[3SΔ20] (*msl-1*$^{m3SΔ20}$/*msl-1*$^{m3SΔ20}$), M1[vCC] (*msl-1*$^{vCC}$/*msl-1*$^{vCC}$), and M1[Δ20vCC] (*msl-1*$^{vCCΔ20}$/*msl-1*$^{vCCΔ20}$).

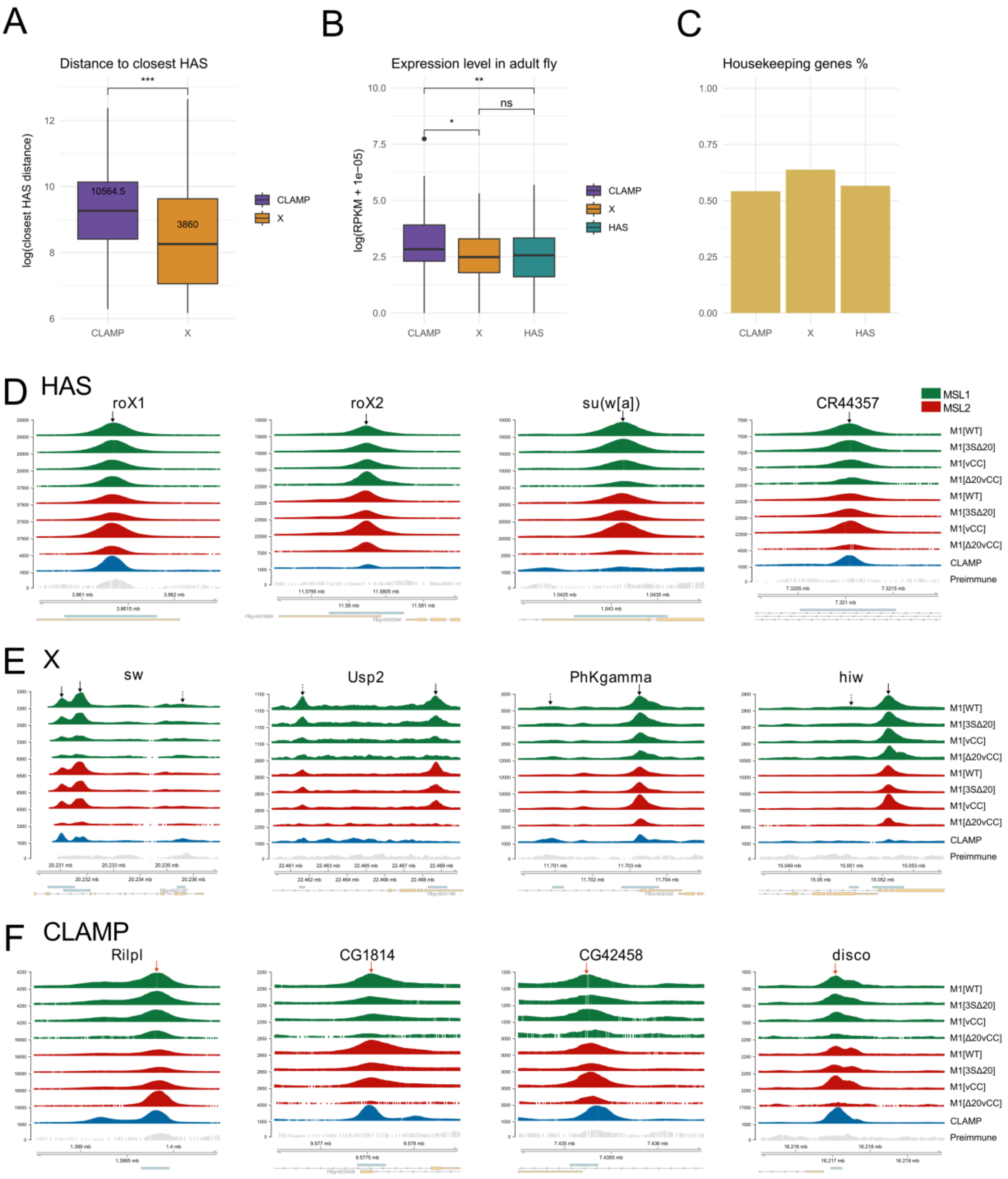


**Fig. S4. Comparison of three groups of MSL1 binding sites (HAS, X, and CLAMP) in adult males.** (**A**) Distribution of distances between CLAMP or X sites to the nearest HAS (only X chromosome sites are shown). Data were compared between groups using the Wilcoxon rank-sum test. (**B**) Gene expression levels (see Methods) of genes associated with each MSL1 group; significance was tested using the Wilcoxon rank-sum test. (**C**) The proportion of housekeeping genes (see Methods) within each group of MSL1 sites. (**D**) Example binding profiles of MSL1, MSL2, and CLAMP at MSL1 sites overlapping HAS; summit positions are indicated with solid arrows. (**E**) Example binding profiles of MSL1, MSL2, and CLAMP at MSL1 sites assigned to the X group; site summits are indicated by dotted arrows, and the nearest HAS summits are indicated by solid arrows. (**F**) Example binding profiles of MSL1, MSL2, and CLAMP at MSL1 sites assigned to the CLAMP group; site summits are indicated by red arrows.

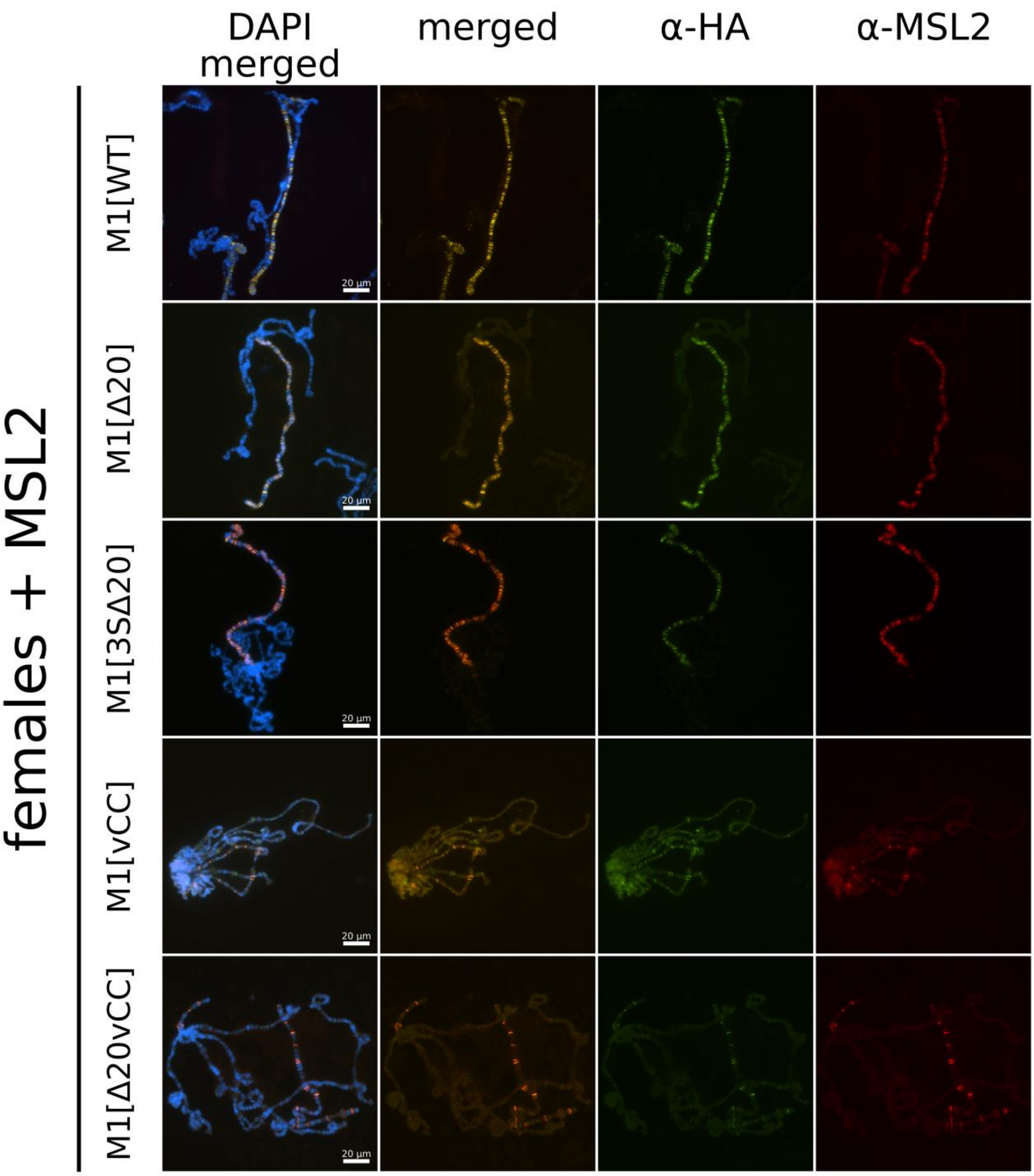


**Fig. S5. Distribution of the MSL complex on the polytene chromosomes from third instar female larvae expressing MSL1* mutants with FLAG-tagged MSL2$^{WT}$.** The panels show immunostaining with a mouse anti-HA antibody (green) and rat anti-MSL2 antibody (red), along with DAPI staining of DNA (blue). The following lines were examined: M1[WT] (*msl-2$^{WT}$/msl-1$^{WT}$*), M1[Δ20] (*msl-2$^{WT}$/msl-1$^{Δ20}$*), M1[3SΔ20] (*msl-2$^{WT}$/msl-1$^{m3SΔ20}$*), M1[vCC] (*msl-2$^{WT}$/msl-1$^{vCC}$*), and M1[Δ20vCC] (*msl-2$^{WT}$/msl-1$^{vCCΔ20}$*).

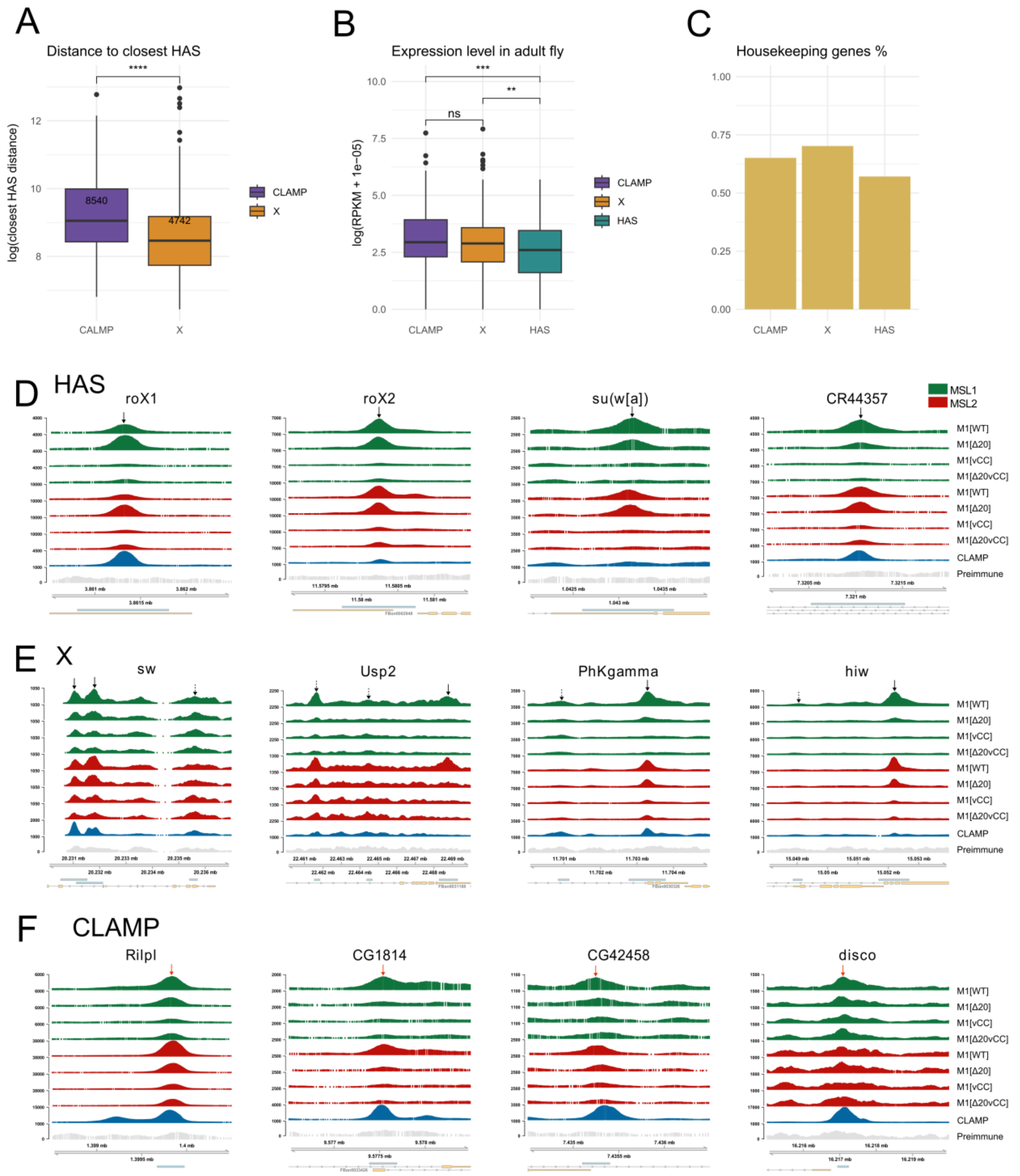


**Fig. S6. Comparison of three groups of MSL1 binding sites (HAS, X, and CLAMP) in adult females.** (**A**) Distribution of distances between CLAMP or X sites to the nearest HAS (only X chromosome sites are shown). Data were compared between groups using the Wilcoxon rank-sum test. (**B**) Gene expression levels (see Methods) of genes associated with each MSL1 group; significance was tested using the Wilcoxon rank-sum test. (**C**) The proportion of housekeeping genes (see Methods) within each group of MSL1 sites. (**D**) Example binding profiles of MSL1, MSL2, and CLAMP at MSL1 sites overlapping HAS; summit positions are indicated by solid arrows. (**E**) Example binding profiles of MSL1, MSL2, and CLAMP at MSL1 sites assigned to the X group; site summits are indicated by dotted arrows, and the nearest HAS summits are indicated by solid arrows. (**F**) Example binding profiles of MSL1, MSL2, and CLAMP at MSL1 sites assigned to the CLAMP group; site summits are indicated by red arrows.
